# L-Lysine production from glucose and chitin monomers using engineered *Vibrio natriegens*

**DOI:** 10.1007/s00253-026-14090-8

**Published:** 2026-11-04

**Authors:** Elly Straube, Thi Van Anh Tran, Anna Faber, Nadine Ihle, Rubén Crespo Blanco, Ha Thanh Le, Georg Fritz, Cláudio J. R. Frazão, Thomas Walther

**Affiliations:** 1 Institute of Natural Materials Technology, TU Dresden, 01062 Dresden, Germany; 2 School of Molecular Sciences, The University of Western Australia, Crawley, 6009 Australia; 3 Forrest Research Foundation, Crawley, 6009 Australia; 4 Oceans Institute, The University of Western Australia, Crawley, 6009 Australia; 5 School of Chemistry and Life Science, Hanoi University of Science and Technology, 1 Dai Co Viet, Hanoi, Vietnam

**Keywords:** L-lysine production, *Vibrio natriegens*, Chitin monomers, Metabolic engineering

## Abstract

**Abstract:**

Despite its industrial importance, microbial L-lysine production has largely been confined to classical producer strains, leaving the fast-growing, non-pathogenic marine bacterium *V. natriegens* mostly unexplored as an emerging biosynthetic platform. In this work, we established an L-lysine-overproducing *V. natriegens* DSM759 strain through a stepwise rational engineering strategy targeting the native biosynthetic pathway. Building on our prior systems-level analysis of the strain’s genetic and regulatory architecture, we identified key metabolic bottlenecks and targeted interventions to relieve them. The central intervention was relief of feedback inhibition on the two key regulatory enzymes, aspartate kinase (AK, *lysC*) and dihydrodipicolinate synthase (DHDPS, *dapA*). Transferring feedback resistance substitutions previously characterized in *E. coli* to conserved positions in the *V. natriegens* DSM759 enzymes produced L-lysine-insensitive variants with kinetic parameters comparable to the corresponding wild-type enzymes. Among the tested configurations, the strain co-expressing *Vn.lysC2* and *Vn.dapA1*:E84T reached the highest L-lysine titer (9.0 ± 0.6 mM) and yield (0.11 ± 0.01 mol_Lys_ mol_Glc_^−1^), whereas overexpression of additional L-lysine pathway genes provided no further benefit. Exploiting the host’s substrate range, we further demonstrated L-lysine synthesis from the chitin-derived amino sugar N-acetylglucosamine (0.09 ± 0.00 mol_Lys_ mol_GlcNAc_^−1^), highlighting the potential to valorize chitin-rich waste streams from the seafood industry. This work establishes a minimal rational strategy for L-lysine biosynthesis in *V. natriegens* DSM759 and positions it as a candidate platform for sustainable amino acid production.

**Key points:**

• *E. coli-derived rational engineering strategies were transferable to V. natriegens*

• *Native pathway deregulation was sufficient to enable L-lysine production*

• *Chitin-derived N-acetylglucosamine supports L-lysine synthesis*

**Supplementary information:**

The online version contains supplementary material available at https://doi.org/10.1007/s00253-026-14090-8.

## Introduction

l‑Lysine is a basic, essential amino acid with central roles in protein synthesis, hormone production, and immune function (Lopez and Mohiuddin 2025; Holeček 2025). Because animals cannot synthesize L-lysine de novo, this amino acid is a critical supplement in human nutrition and livestock feed (Cheng et al. 2018). Beyond its nutritional role, L-lysine is used in pharmaceutical and cosmetic formulations and serves as a precursor for bio-based chemicals such as putrescine, ε-poly-L-lysine, and nylon monomers (Xu et al. 2016b; Jorge et al. 2017; Kumar et al. 2019; Li et al. 2022; Wu et al. 2024). To meet the increasing global demand, industrial L-lysine production relies predominantly on microbial fermentation using extensively engineered *Escherichia coli* and *Corynebacterium glutamicum* strains (Liu et al. 2022; Wu et al. 2024). Key strain engineering strategies include eliminating feedback inhibition of the rate-limiting enzymes and overexpressing biosynthetic genes in the L-lysine pathway (Becker et al. 2011; Xu et al. 2016a), optimizing precursor and cofactor supply, and suppressing competing pathways. System-biology approaches, including genome-scale and enzyme-constrained modeling, have further guided the identification of pathway bottlenecks (Ye et al. 2020). Industrial processes typically employ fed-batch fermentation to balance biomass formation with amino acid synthesis (Kiss and Stephanopoulos 1991; Ying et al. 2014), resulting in peak titers of 120–190 g L^−1^. Despite these advances, challenges remain, including minimization of by-products, simpler downstream purification, lower substrate costs, and improved robustness under non-sterile conditions (Liu et al. 2022; Yin et al. 2024; Wu et al. 2024).

**Fig. 1:**
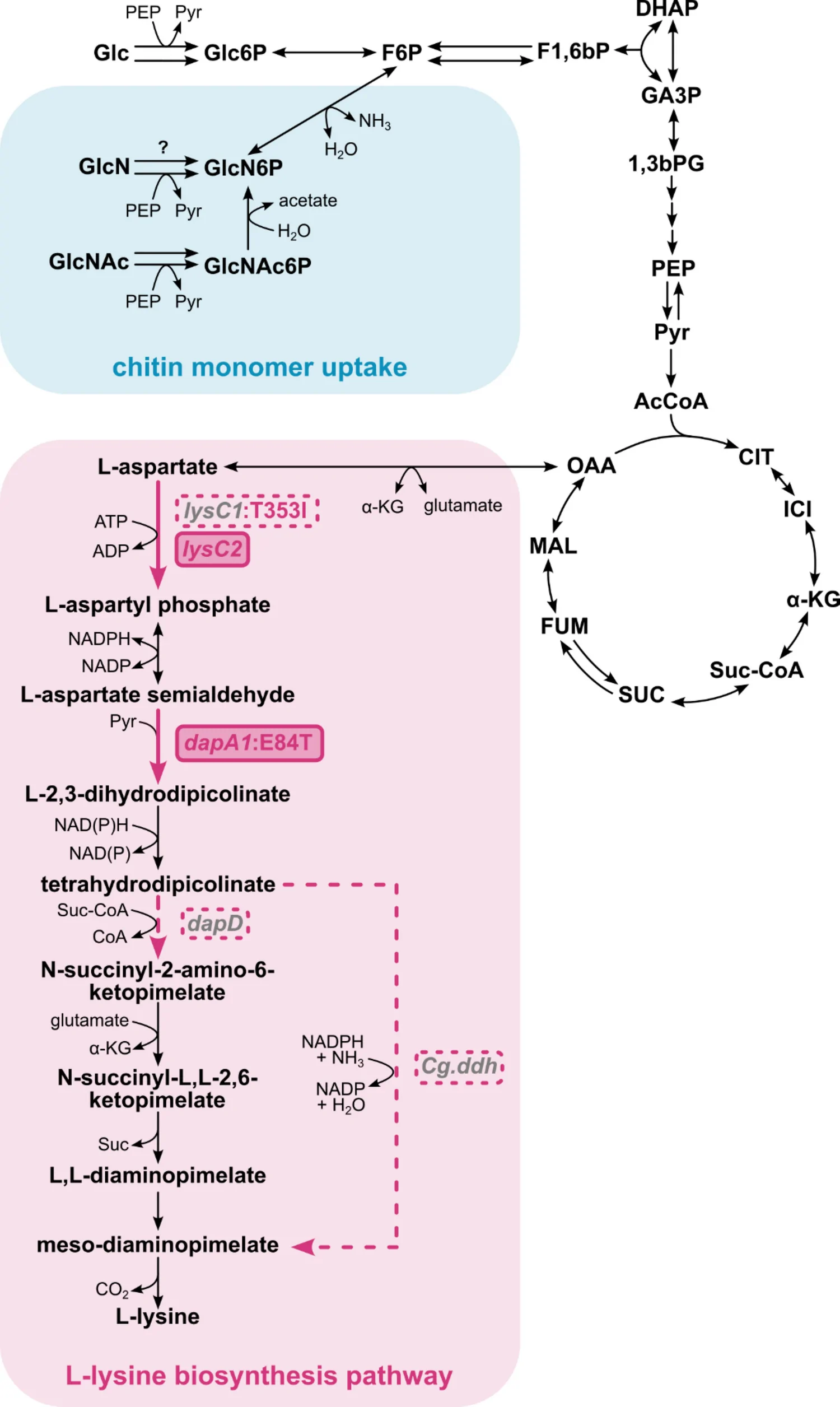
Biosynthesis of L-lysine from glucose and chitin monomers and applied engineering strategies for creation of a *V. natriegens* L-lysine overproducer strain. Glucose uptake, glycolysis, and TCA cycle are shown in simplified form, while chitin monomer uptake and L-lysine biosynthesis pathways are depicted in detail, including side products. Glucosamine (GlcN) import remains uncharacterized and is therefore indicated with a question mark. Tested plasmid-based (over-)expression strategies are indicated in pink dashed lines, whereas solid pink marks the overexpressed genes and reactions in the best-performing producer. Abbreviations: Glc, glucose; Glc6P, glucose-6-phosphate; F6P, fructose-6-phosphate; F1,6bP, fructose-1,6-bisphosphate; DHAP, dihydroxyacetone phosphate; GA3P, glyceraldehyde-3-phosphate; 1,3bPG, 1,3-bisphosphoglycerate; PEP, phosphoenolpyruvate; Pyr, pyruvate; AcCoA, acetyl-coenzyme A; CIT, citrate; ICI, isocitrate; α-KG, α-ketoglutarate; Suc-CoA, succinyl-coenzyme A; SUC, succinate; FUM, fumarate; MAL, malate; OAA, oxaloacetate; GlcN, glucosamine; GlcN6P, glucosamine-6-phosphate; GlcNAc, N-acetylglucosamine; GlcNAc6P, N-acetylglucosamine-6-phosphate. Gene names are written in gray, bold italic font. *lysC1*(:T353I) – gene encoding native L-lysine-sensitive, mono-functional aspartate kinase, and engineered insensitive mutant; *lysC2*, gene encoding native L-lysine-insensitive, mono-functional aspartate kinase; *dapA1*(:E84T), gene encoding native L-lysine-sensitive dihydrodipicolinate synthase and engineered insensitive mutant; *dapD*, gene encoding native diaminopimelate decarboxylase; *Cg.ddh*, gene encoding diaminopimelate dehydrogenase from *C. glutamicum*

*Vibrio natriegens* has attracted increasing attention as a microbial production chassis because of its exceptionally high growth and substrate uptake rates, which promise markedly shorter fermentation processes and higher space–time yields (Hoff et al. 2020; Thoma and Blombach 2021; Hädrich et al. 2025). Over the past decade, its potential has been explored for the biosynthesis of diverse compounds, including organic and amino acids (Hoffart et al. 2017; Thoma et al. 2022; Wu et al. 2023), biopolymers (Dalia et al. 2017; Politan et al. 2025; Faber et al. 2026), pigments (Ellis et al. 2019; Wang et al. 2020), and short-chain alcohols (Erian et al. 2020; Zhang et al. 2021). In parallel, a steadily expanding genetic toolbox—comprising standardized expression parts (Stukenberg et al. 2021; Faber et al. 2025) and multiple genome-editing methods (Dalia et al. 2017; Stukenberg et al. 2022) has improved its tractability for metabolic engineering and positions *V. natriegens* as a strong candidate for future biotechnological applications. Building on this foundation, we recently characterized the genomic organization and regulation of the L-lysine pathway in *V. natriegens*: As a member of the aspartate amino acid family, L-lysine is synthesized from l-aspartate through nine enzymatic steps via the diaminopimelate (DAP) pathway (Fig. 1). L-lysine exerts regulatory control over its own biosynthesis at both the enzymatic and transcriptional levels. It strongly inhibits two key enzymes, monofunctional aspartate kinase (AK, Vn.LysC1) and dihydrodipicolinate synthase (DHDPS, Vn.DapA1), and represses transcription of *Vn.lysC1* and *Vn.dapD*. The genetic organization and regulatory architecture of this pathway closely mirror those of *E. coli*, highlighting conserved mechanisms of feedback control. Unlike *E. coli*, *V. natriegens* also encodes a second, intrinsically feedback-resistant monofunctional AK isozyme (Vn.LysC2), which may provide a native bypass around one of the primary metabolic bottlenecks (Straube et al. 2026). Nonetheless, dual-level regulation implies that multiple control points must be addressed to achieve efficient L-lysine overproduction. Proof-of-concept studies using transcription factor-based biosensors have isolated L-lysine-secreting mutants producing up to 1 mM extracellularly, confirming pathway accessibility despite low initial titers (Stella et al. 2021). A subsequent study demonstrated increased L-lysine biosynthesis in *V. natriegens* DSM759 through genomic integration of heterologous genes from *C. glutamicum* (Nghiem et al. 2025). However, the simultaneous introduction of multiple pathway components in that work precluded quantitative assessment of their individual contributions and thus provided limited guidance for targeted flux optimization.

Here, we engineer L-lysine production in *V. natriegens* DSM759 by rationally optimizing its native biosynthetic pathway on the basis of our prior pathway analysis. First, feedback inhibition of key enzymes was alleviated by creating L-lysine-insensitive mutants of the native AK and DHDPS isozymes. The effects of these deregulated variants on L-lysine production were then systematically analyzed through plasmid-based overexpression of the corresponding genes. To complement the alleviation of enzymatic regulation and further address potential rate-limiting steps, transcriptional repression of *Vn.dapD,* which encodes tetrahydrodipicolinate succinylase (DapD), was targeted. This was approached in two ways: plasmid-based overexpression of the native gene or introduction of the heterologous diaminopimelate dehydrogenase (DapDH), which bypasses the native reaction sequence. Finally, leveraging the metabolic versatility of *V. natriegens* DSM759, we examined L-lysine production from chitin-derived amino sugars as alternative feedstocks, illustrating the host’s potential for valorization of chitin-rich crustacean shell waste. Together, these interventions establish a stepwise rational framework for L-lysine biosynthesis in *V. natriegens* DSM759 and highlight the host’s potential as a fast-growing platform for sustainable amino-acid production.

## Materials and methods

### Reagents and chemicals

Chemicals, solvents, and oligonucleotides were purchased from Sigma-Aldrich (Darmstadt, Germany), unless stated otherwise. Plasmid DNA purification and gel extraction kits as well as restriction enzymes were purchased from NEB (Frankfurt am Main, Germany). Sanger sequencing was carried out by Eurofins (Ebersberg, Germany).

### Strains and plasmids

All strains and plasmids used in this study are listed in Tables 1 and 2.

**Table 1:** *Escherichia coli* and *Vibrio natriegens* strains used in this study

Strain	Genotype	Reference/origin
NEB® 5-alpha	*E. coli fhuA2*::IS2 Δ(*mmuP*-*mhpD*)169 Δ*phoA8 glnX44* ϕ80d[Δ*lacZ58*(M15)] *rfbD1 gyrA96 luxS11 recA1 endA1 rphWT thiE1 hsdR17*	NEB™
NEB® stable	*E. coli F' proA*^+^*B*^+^ *lacI*^*q*^ ∆(*lacZ*)M15 *zzf*::Tn10 (Tet^R^)/∆(*ara-leu*) 7697 *araD139 fhuA* ∆*lacX74 galK16 galE15* e14- Φ*80dlacZ*∆*M15 recA1 relA1 endA1 nupG rpsL* (Str^R^) *rph spoT1* ∆(*mrr-hsdRMS-mcrBC*)	NEB™
BL21 (DE3)	*E. coli fhuA2* [lon] *ompT gal* (λ DE3) [dcm] ∆*hsdS*	NEB™
*V. natriegens*	*V. natriegens* DSM759 (ATCC14048) Δ*dns*	Stukenberg et al. (2021)

**Table 2:** Plasmids used in this study

Plasmid	Relevant characteristics	Reference/origin
In vitro* studies*		
pET28a(+)	*f1* origin, Kan^R^, P_T7_	Novagen™
pET28-*Vn.lysC1*	pET28a(+) derivative with N-terminal his-tagged *Vn.lysC1*	Straube et al. (2026)
pET28-*Vn.lysC2*	pET28a(+) derivative with N-terminal his-tagged *Vn.lysC2*	Straube et al. (2026)
pET28-*Vn.lysC1*:E251K	pET28a(+) derivative with N-terminal his-tagged *Vn.lysC1*:E251K	This study
pET28-*Vn.lysC1*:V340A	pET28a(+) derivative with N-terminal his-tagged *Vn.lysC1*:V340A	This study
pET28-*Vn.lysC1*:D341P	pET28a(+) derivative with N-terminal his-tagged *Vn.lysC1*:D341P	This study
pET28-*Vn.lysC1*:T353I	pET28a(+) derivative with N-terminal his-tagged *Vn.lysC1*:T353I	This study
pET28-*Vn.dapA1*	pET28a(+) derivative with N-terminal his-tagged *Vn.dapA1*	Straube et al. (2026)
pET28-*Vn.dapA1*:E84T	pET28a(+) derivative with N-terminal his-tagged *Vn.dapA1*:E84T	This study
In vivo *l-lysine production*		
pMBI-GFP dropout	pMBI origin, Kan^R^(Vn), *lacI* under P_*lacI*_, transcriptional unit: P_tac_ – RBS (B0032) – GFP dropout – terminator (B0015)	This study
pMBI	empty pMBI derivative (without GFP dropout)	This study
pMBI_*Vn.lysC1*	pMBI derivative carrying *Vn.lysC1*	This study
pMBI_*Vn.lysC1*:T353I	pMBI derivative carrying *Vn.lysC1*:T353I	This study
pMBI_*Vn.lysC2*	pMBI derivative carrying *Vn.lysC2*	This study
pMBI_*Vn.dapA1*	pMBI derivative carrying *Vn.dapA1*	This study
pMBI_*Vn.dapA1*:E84T	pMBI derivative carrying *Vn.dapA1*:E84T	This study
pMBI_*Vn.lysC1*:T353I_*Vn.dapA1*:E84T	pMBI derivative carrying *Vn.lysC1*:T353I and *Vn.dapA1*:E84T	This study
pMBI_*Vn.lysC2*_*Vn.dapA1*:E84T	pMBI derivative carrying *Vn.lysC2* and *Vn.dapA1*:E84T	This study
pMBI_*Vn.lysC2*_*Vn.dapA1*:E84T_*Vn.dapD*(B0032)	pMBI derivative carrying *Vn.lysC2, Vn.dapA1*:E84T and *Vn.dapD* (*Vn.dapD* preceded by RBS B0032)	This study
pMBI_*Vn.lysC2*_*Vn.dapA1*:E84T_*Vn.dapD*(B0064)	pMBI derivative carrying *Vn.lysC2, Vn.dapA1*:E84T and *Vn.dapD* (*Vn.dapD* preceded by RBS B0064)	This study
pMBI_*Vn.lysC2*_*Vn.dapA1*:E84T_*Cg.ddh*(B0032)	pMBI derivative carrying *Vn.lysC2, Vn.dapA1*:E84T and *Cg.ddh* (*Cg.ddh* preceded by RBS B0032)	This study
pMBI_*Vn.lysC2*_*Vn.dapA1*:E84T_*Cg.ddh*(B0064)	pMBI derivative carrying *Vn.lysC2, Vn.dapA1*:E84T and *Cg.ddh* (*Cg.ddh* preceded by RBS B0064)	This study
pMBI_*Vn.lysC2*_*Vn.dapA1*:E84T_*Cg.ddh*:CO(B0032)	pMBI derivative carrying *Vn.lysC2, Vn.dapA1*:E84T and codon-optimized variant of *Cg.ddh* (*Cg.ddh*:CO preceded by RBS B0032)	This study
pMBI_*Vn.lysC2*_*Vn.dapA1*:E84T_*Cg.ddh*:CO(B0064)	pMBI derivative carrying *Vn.lysC2, Vn.dapA1*:E84T and codon-optimized variant of *Cg.ddh* (*Cg.ddh*:CO preceded by RBS B0064)	This study

### Media

LB medium (LB Broth (Luria/Miller), Carl Roth) and LB agar plates (LB agar (Luria/Miller), Carl Roth) were used for cloning procedures, protein production, and recovery of cells from glycerol stocks (25% v v^−1^, stored at − 80 °C). For work with *V. natriegens*, LB medium was supplemented with v2 salts (204 mM NaCl, 4.2 mM KCl, and 23.14 mM MgCl_2_; LBv2) (Weinstock et al. 2016). Where required, kanamycin sulfate was added at 50 mg L^−1^ for *E. coli* and 200 mg L^−1^ for *V. natriegens*.

For in vivo L-lysine production in shake-flask experiments, an adapted sulfur-limited VN mineral medium was used (Hoffart et al. 2017), containing 100 mM of carbon source (glucose, glucosamine, or N-acetylglucosamine), 150 mM NH_4_Cl, 15 g L^−1^ NaCl, 1 g L^−1^ KH_2_PO_4_, 1 g L^−1^ K_2_HPO_4_, 2 mM MgCl_2_, 10 mg L^−1^ CaCl_2_, 16.4 mg L^−1^ FeSO_4_ · 7 H_2_O, and trace elements (10 mg L^−1^ MnSO_4_ · H_2_O, 0.3 mg L^−1^ CuSO_4_ · 5 H_2_O, 1 mg L^−1^ ZnSO_4_ · 7 H_2_O, and 0.02 mg L^−1^ NiCl_2_ · 6 H_2_O). The medium was buffered with 200 mM MOPS.

### Side-directed mutagenesis, gene expression, and protein purification for in vitro enzyme studies

Site-directed mutagenesis to generate L-lysine-resistant AK and DHDPS isozymes was performed by inverse PCR (Zheng et al. 2004) using the primer pairs listed in Table S1. Each reaction contained 6 ng of the pET28 plasmid carrying the respective wild-type enzyme as template. PCR was carried out with Q5 polymerase (NEB), using a reduced cycle number of 18. Residual template DNA was removed by *Dpn*I digestion (NEB), and the resulting plasmids were transformed into chemically competent NEB® 5-alpha (NEB) and verified by Sanger sequencing. All constructed plasmids are listed in Table 2.

Gene expression and protein purification were carried out as previously described by Straube et al. (2026).

### AK and DHDPS enzymatic assay

Enzymatic assays to determine kinetic parameters and L-lysine resistance of wild-type and mutant AK and DHDPS enzymes were performed as previously described by Straube et al. (2026).

### Plasmid construction for in vivo L-lysine production

All plasmids constructed and used for in vivo L-lysine biosynthesis are listed in Table 2. In this study, we designed and assembled the pMBI plasmid backbone, which contains a GFP dropout cassette serving as a placeholder for insertion of target genes within a single transcriptional unit (pMBI-GFP dropout). The backbone was assembled using Golden Gate cloning following the strategy of Stukenberg et al. (2021).

Expression constructs were generated by replacing the GFP dropout cassette with one, two, or three coding sequences along with their respective ribosome binding sites (RBSs). RBSs were assigned according to gene position within the operon—B0032 (first gene), B0033 (second), and B0032/B0064 (third)—and were sourced from the Vnat Collection (Stukenberg et al. 2021; Faber et al. 2025). Constructs were assembled using isothermal assembly (NEBuilder® HiFi DNA Assembly Master Mix, NEB) following the manufacturer’s instructions. Target genes were amplified by PCR from the respective pET28 plasmids or from *V. natriegens* DSM759 chromosomal DNA using Q5 polymerase (NEB) (GenBank accession numbers: *Vn.lysC1* - ALR14506.1; *Vn.lysC2* - ALR14655.1; *Vn.dapA1* - ALR14900.1), with primers listed in Table S1. The pMBI-GFP dropout backbone was linearized by *Bbs*I digestion (NEB), and fragments were purified by gel extraction (Monarch® Spin DNA Gel Extraction Kit, NEB) prior to assembly.

An empty pMBI vector was generated as a control by PCR amplification of the digested backbone to create overlapping regions, followed by self-ligation using NEBuilder® HiFi DNA Assembly Master Mix.

Plasmids carrying the *Cg.ddh* gene and/or RBS B0032 upstream of the third gene of the transcriptional unit were generated by restriction-ligation-based cloning. The backbone (pMBI_*Vn.lysC2*_*Vn.dapA1*:E84T) and the *Vn.dapD* (GenBank accession number: ALR14800.1) or *Cg.ddh* (GenBank accession number: BAC00011.1) inserts were amplified by PCR using Q5 polymerase. Wild-type *Cg.ddh* was amplified from *Corynebacterium glutamicum* DSM20300 chromosomal DNA, and the codon-optimized variant was obtained from a synthetic gene (Azenta). PCR primers introduced *Nde*I and *Eco*RI (for insertion of *Vn.dapD*) or *Nde*I and *Sal*I restriction sites (for insertion of *Cg.ddh*) at the 5′ and 3′ ends (Table S1). Both backbone and inserts were digested with *Nde*I and *Eco*RI/*Sal*I (NEB), purified by gel extraction, and ligated using T4 DNA ligase (NEB) according to the manufacturer’s protocol.

All constructed pMBI vectors were transformed into chemically competent NEB® 5-alpha or NEB® stable (NEB) and verified by Sanger sequencing.

### Shake-flask cultivation for L-lysine production

Shake-flask cultivations were performed at 30 or 37 °C, 220 rpm in an orbital shaker (Ecotron, Infors). For each experiment, *V. natriegens* cells were freshly transformed with the respective pMBI plasmids following the protocol of Stukenberg et al. (2021). For precultures, single colonies were inoculated into 5-mL LBv2 medium in 100-mL shake flasks and incubated overnight at 30 °C. Cells were harvested by centrifugation (4500 *g*, 10 min, room temperature), washed with 9 g L^−1^ NaCl, and resuspended to inoculate the main cultures at a starting OD_600 nm_ of 0.2. Main cultures were grown in 25 mL sulfur-limited VN mineral medium in 250 mL baffled shake flasks at 37 °C. When cultures reached an OD_600 nm_ of 0.6–0.8, gene expression was induced by addition of 1 mM IPTG. Samples were collected at regular intervals, centrifuged (13,000 *g*, 5 min, room temperature), and the supernatant stored at − 20 °C until further analysis.

### Analytical methods

Substrate (glucose, N-acetylglucosamine) and metabolite concentrations (acetate, pyruvate, and L-lysine) were quantified by HPLC. Supernatants (1 mL) were filtered through 0.22-µm regenerated cellulose filters (VWR) and transferred into 1.5-mL HPLC vials (VWR). Samples were analyzed on an AZURA® 862 bar HPLC system (Knauer), equipped with a P 6.1 L pump (LPG), AS 2.1 L autosampler (set to 6 °C), CT 2.1 column oven, RI detector (RID 2.1 L; set to 45 °C), and UV/Vis detector (MWD 2.1 L). The injection volume was 20 µL.

Sugars and organic acids were separated on a Rezex™ ROA-Organic Acid H^+^ (8%) column (300 × 7.8 mm; Phenomenex) with a SecurityGuard™ Carbo-H^+^ precolumn (4 × 3 mm; Phenomenex). Elution was performed with 0.5 mM H_2_SO_4_ at 0.5 mL min^−1^, and the column temperature was maintained at 80 °C.

L-lysine was quantified using a Chirex 3126 (D)-penicillamine column (150 × 4.6 mm; Phenomenex) protected by a SecurityGuard™ AQ C18 cartridge (4 × 3 mm; Phenomenex). Elution was performed with 2 mM CuSO_4_ as mobile phase at 0.5 mL min^−1^, and the column temperature was maintained at 25 °C. To ensure the removal of late-eluting or otherwise retained compounds and to maintain long-term column performance, each chromatographic run included a methanol ramp in the mobile phase up to 5% (v/v), followed by a return to 0%.

### Computational methods

Pairwise local sequence alignments were performed to determine whether amino acid residues in *V. natriegens* AK and DHDPS corresponding to L-lysine-resistant positions in *E. coli* were conserved. Amino acid sequences of the *E. coli* wild-type enzymes and the respective *V. natriegens* homologs were obtained from the KEGG database (Kanehisa and Goto 2000) and aligned using the EMBOSS Water algorithm (Smith-Waterman) via the EMBOSS web server (Madeira et al. 2024). Default parameters were used.

The sequence of *Corynebacterium glutamicum* diaminopimelate dehydrogenase *(Cg.ddh)* was manually codon-optimized for expression in *V. natriegens*. Rare codons throughout the gene were identified and optimized using the Graphical Codon Usage Analyser (GCUA; gcua.schoedl.de) based on the *V. natriegens* codon-usage table from (Lee et al. 2019). Additionally, the first 27 bases of the sequence were analyzed using RNAfold (ViennaRNA package) (Gruber et al. 2008) to predict potential secondary structures, and adjusted as needed to minimize stable base-pairing. The final codon-optimized gene was synthesized by Azenta, Inc., and the nucleotide sequence is provided in the Supplementary Material (Online Resource Text S1).

## Results

### Construction of L-lysine-resistant AK and DHDPS enzymes

To relieve feedback inhibition of the L-lysine biosynthetic pathway, the key regulatory enzymes AK and DHDPS of *V. natriegens* were engineered by site-directed mutagenesis.

**Fig. 2:**
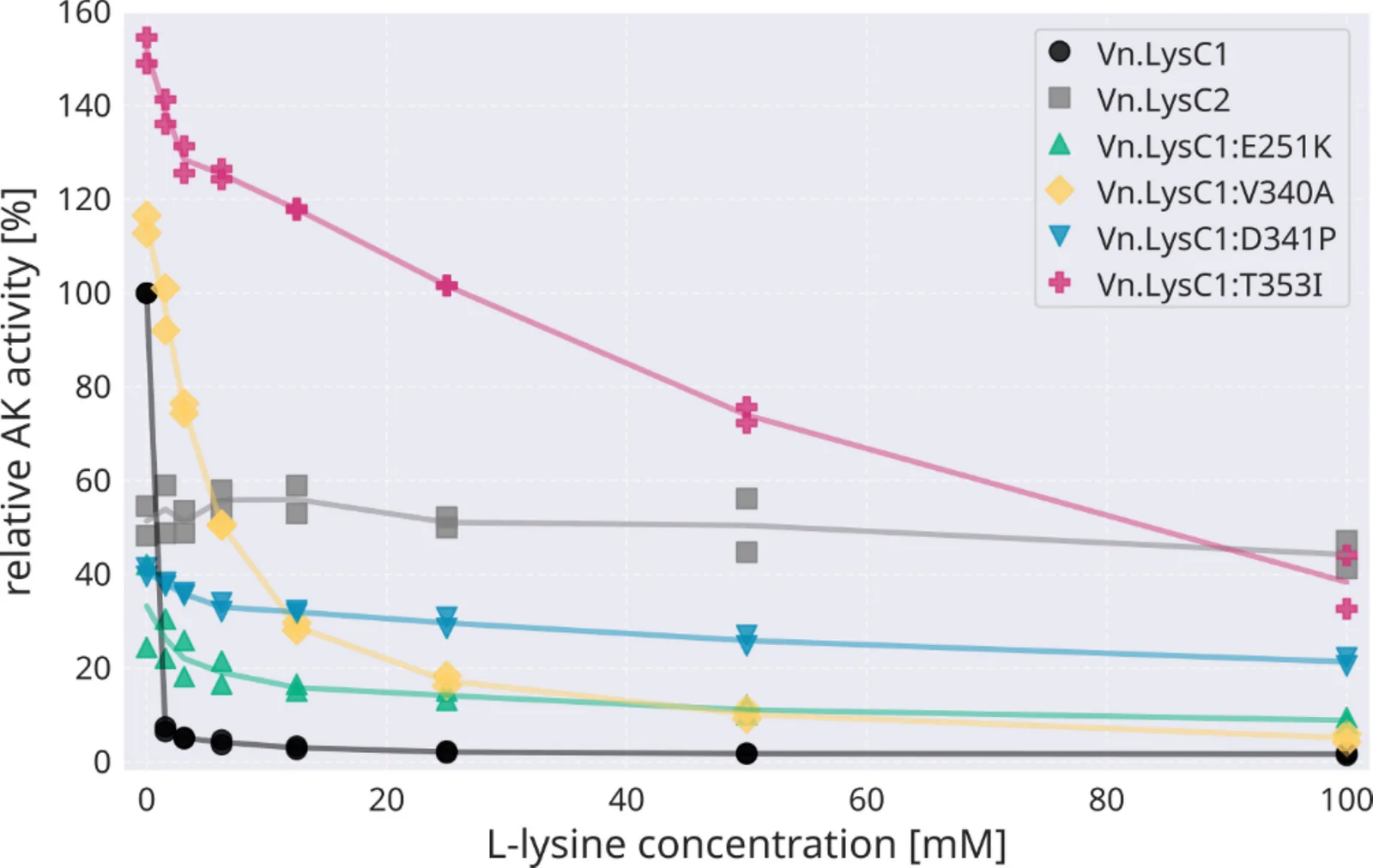
Construction of a L-lysine-insensitive Vn.LysC1 mutant. The relative specific activities of native AK isozymes Vn.LysC1 and Vn.LysC2, and single amino acid mutants of Vn.LysC1, were determined with 50 mM l-aspartate as substrate in the presence of 0–100 mM L-lysine. Activities were normalized to the activity of Vn.LysC1 measured in the absence of L-lysine (v_max_ = 14.43 ± 2.81 U mg^−1^; pH 7.5; 37 °C; individual data points represent biological replicates; lines connect the means at each L-lysine concentration (*n* = 2))

AK catalyzes the first step of the L-lysine biosynthetic pathway, the phosphorylation of l-aspartate to l-aspartyl phosphate (Fig. 1). Two monofunctional AK isozymes have previously been characterized in *V. natriegens*: Vn.LysC1, which is strongly inhibited by L-lysine, and Vn.LysC2, which is intrinsically insensitive to L-lysine (Straube et al. 2026). Sequence analysis revealed that Vn.LysC1 shares high sequence similarity with Ec.LysC and retains conserved ACT regulatory domains implicated in allosteric regulation (Kotaka et al. 2006), whereas Vn.LysC2 exhibits lower sequence similarity and lacks these domains (Straube et al. 2026). Accordingly, two complementary routes were considered toward a monofunctional AK that is no longer subject to feedback inhibition by L-lysine: direct utilization of the intrinsically insensitive isozyme Vn.LysC2, or engineering of feedback-resistant Vn.LysC1 variants. As the former strategy required no enzyme engineering, subsequent work focused on the construction and characterization of Vn.LysC1 mutants. Four amino acid substitutions previously reported to reduce L-lysine sensitivity in Ec.LysC were selected for transfer to the corresponding conserved residues of Vn.LysC1: E250K (Kikuchi et al. 1999), V339A (Chen et al. 2011b), D340P (Wang et al. 2016), and T352I (Kikuchi et al. 1999; Ogawa-Miyata et al. 2001). The corresponding Vn.LysC1 variants (E251K, V340A, D341P, and T353I) were generated by site-directed mutagenesis using inverse PCR. The L-lysine sensitivity of the resulting variants was determined by in vitro enzymatic assays and compared with those of wild-type Vn.LysC1 and Vn.LysC2 (Fig. 2). In the absence of L-lysine, Vn.LysC1:V340A and Vn.LysC1:T353I showed elevated baseline activities (115% and 152% of Vn.LysC1 wild type), whereas Vn.LysC1:E251K and Vn.LysC1:D341P retained only 33–41% of wild-type activity. Under the same conditions, Vn.LysC2 exhibited 51% of the activity of wild-type Vn.LysC1. Upon exposure to increasing L-lysine concentrations (2–100 mM), wild-type Vn.LysC1 displayed strong, concentration-dependent inhibition, retaining only 2–7% residual activity, whereas Vn.LysC2 remained largely unaffected, maintaining 44–56% residual activity across all tested concentrations. All four engineered Vn.LysC1 variants showed reduced L-lysine sensitivity relative to wild-type, with T353I exhibiting the most pronounced improvement (38% residual activity at 100 mM L-lysine). Kinetic characterization revealed comparable K_M_ values for l-aspartate among Vn.LysC1, Vn.LysC1:T353I, and Vn.LysC2 (5.35 ± 0.40, 4.29 ± 0.87, and 5.52 ± 0.61 mM, respectively), whereas Vn.LysC1:T353I exhibited a higher v_max_ (26.46 ± 1.80 U mg^−1^) and Vn.LysC2 a lower v_max_ (7.64 ± 1.67 U mg^−1^) than Vn.LysC1 (14.43 ± 2.81 U mg^−1^; Table 3). Thus, the reduced L-lysine sensitivity of T353I was not accompanied by an apparent loss of catalytic activity under the conditions tested. Based on these results, Vn.LysC1:T353I and Vn.LysC2 were selected for subsequent in vivo evaluation as the engineered feedback-resistant and naturally L-lysine-insensitive AK variants, respectively.

**Table 3:** Kinetic parameters of AK and DHDPS enzymes

Enzymatic activity	Enzyme name	v_max_ [U mg^−1^]	K_M_ [mM]	Reference
AK^a^	Vn.LysC1	14.43 ± 2.81	5.35 ± 0.40	Straube et al. (2026)
	Vn.LysC2	7.64 ± 1.67	5.52 ± 0.61	Straube et al. (2026)
	Vn.LysC1:T353I	26.46 ± 1.80	4.29 ± 0.87	This study
DHDPS^b^	Vn.DapA1	19.18 ± 4.20	0.20 ± 0.01	Straube et al. (2026)
	Vn.DapA1:E84T	14.85 ± 2.33	0.19 ± 0.00	This study

Experiments were carried out at pH 7.5 and 37 °C. Experimental data was fitted to the Michaelis–Menten model to determine kinetic parameters; data are presented as mean ± SD; *n* = 3 biological replicates

^a^Kinetic parameters of AK activity were determined on 0.012–50 mM l-aspartate as substrate

^b^Kinetic parameters of DHDPS activity were determined on 0.001–1 mM l-aspartate semialdehyde as substrate

**Fig. 3:**
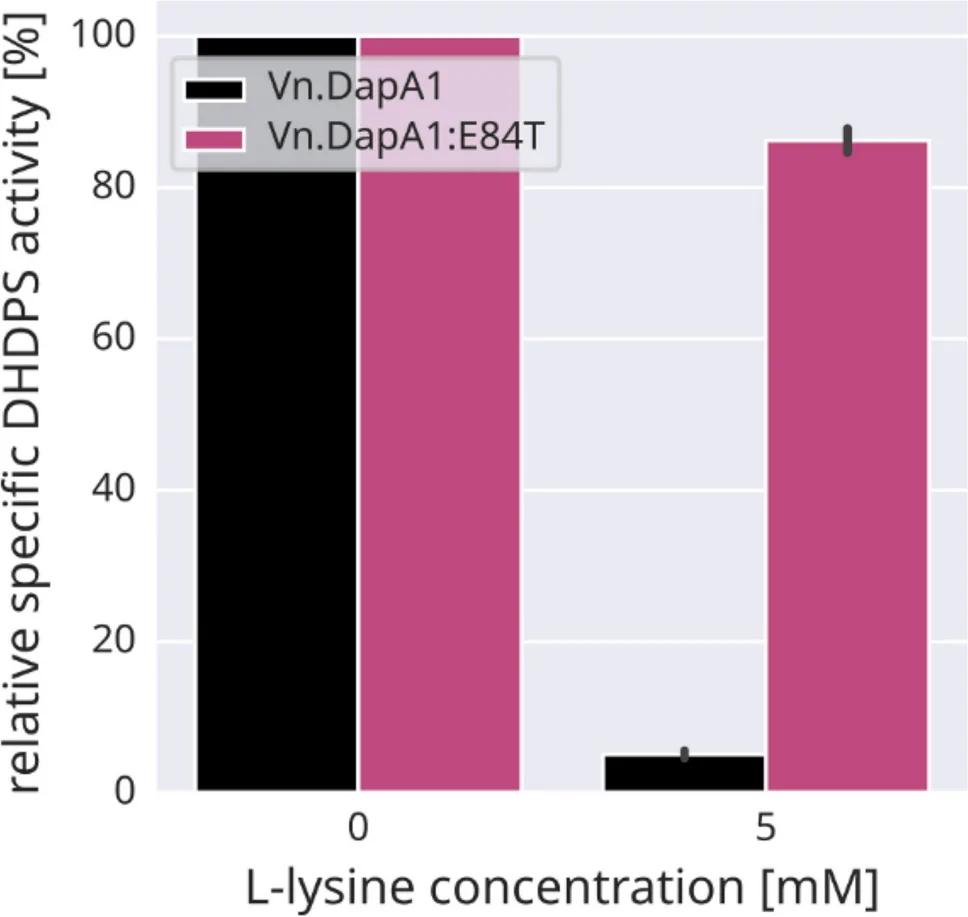
Construction of a L-lysine-insensitive Vn.DapA1 mutant. The relative specific activities of native DHDPS Vn.DapA1 and single amino acid mutant Vn.DapA1:E84T were measured with 0.5 mM ASA as substrate in the presence of 0 and 5 mM L-lysine. Activities were normalized to the activity in the absence of L-lysine (pH 7.5; 37 °C; data are presented as mean ± SD; *n* = 3 biological replicates)

We next turned to DHDPS, the second regulatory enzyme of the L-lysine biosynthetic pathway. DHDPS catalyzes the condensation of l-aspartate semialdehyde (ASA) and pyruvate to form l−2,3-dihydrodipicolinate (Fig. 1). Two DHDPS isozymes have previously been identified in *V. natriegens*: Vn.DapA1 exhibits approximately 270-fold higher specific activity than Vn.DapA2 and was therefore proposed as the major functional isozyme (Straube et al. 2026). Because Vn.DapA1 is also strongly inhibited by L-lysine (Straube et al. 2026), it was selected for engineering. Based on a previously described feedback-resistant Ec.DapA variant (Geng et al. 2013), the conserved residue E84 of *Vn.dapA1* was substituted with l-threonine by site-directed mutagenesis using inverse PCR. The resulting Vn.DapA1:E84T variant retained 86% residual activity in the presence of 5 mM L-lysine, whereas wild-type Vn.DapA1 retained only 5% activity (Fig. 3). Kinetic characterization showed that Vn.DapA1:E84T retained a K_M_ of 0.19 ± 0.00 mM and a v_max_ of 14.85 ± 2.33 U mg^−1^, compared with 0.20 ± 0.01 mM and 19.18 ± 4.20 U mg^−1^, respectively, for wild-type Vn.DapA1 (Table 3). Based on these results, Vn.DapA1:E84T was selected for subsequent in vivo evaluation.

### Effects of feedback-resistant AK and DHDPS overexpression on L-lysine production

**Fig. 4:**
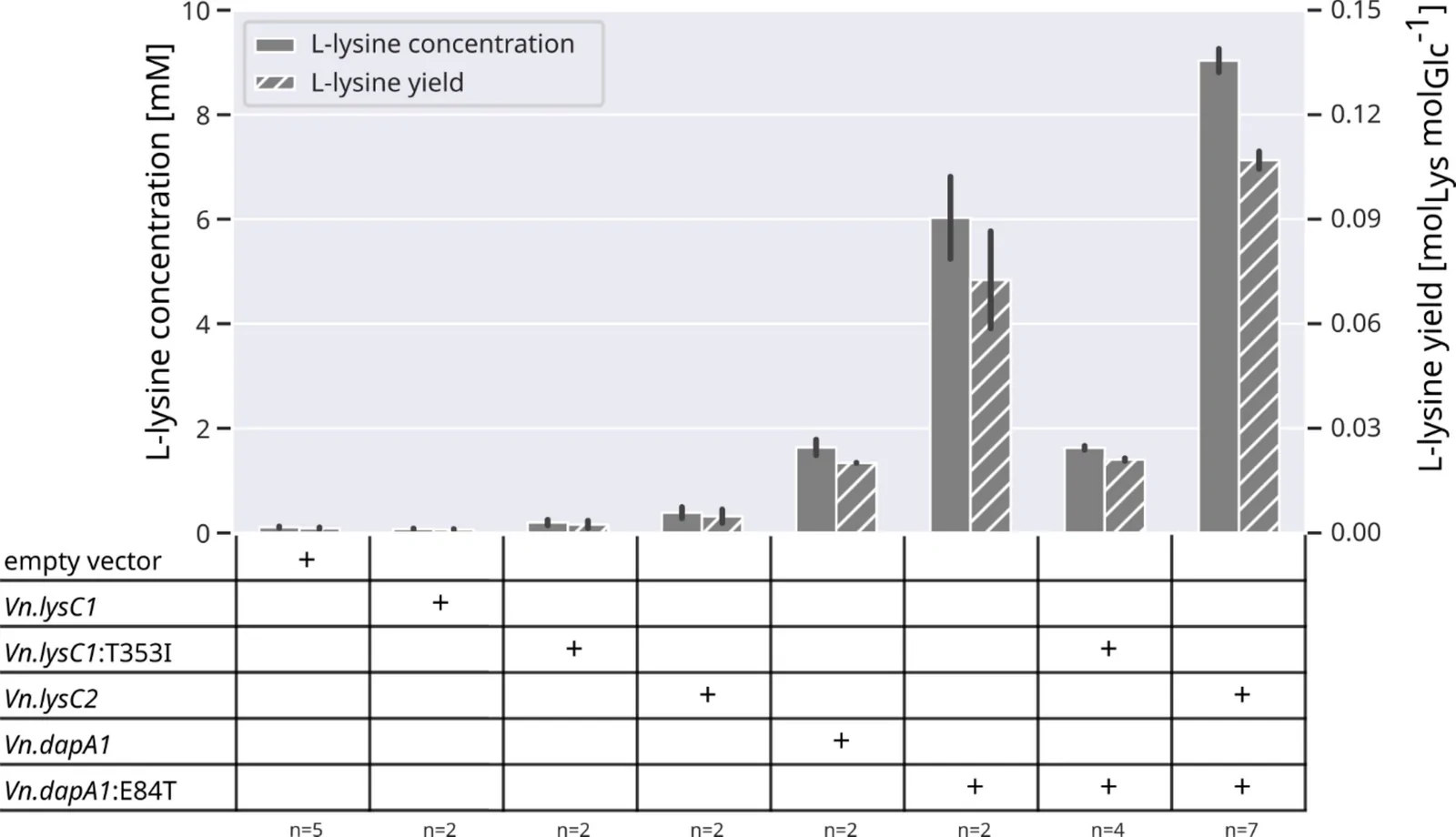
Plasmid-based overexpression of L-lysine-insensitive AK and DHDPS-encoding genes increases L-lysine production in *V. natriegens*. L-lysine concentration after 24 h and L-lysine yield are shown for *V. natriegens* DSM759 ∆*dns* strains harboring different pMBI vectors during growth on glucose. The table below the plot indicates the gene variant expressed in each strain (“+”) (S-limited VN mineral medium with 20 g L^−1^ glucose and 150 mM NH_4_Cl; 1 mM IPTG; 37 °C; 220 rpm; data are presented as mean ± SD; the number of biological replicates (*n*) is indicated for each condition)

To assess the impact of L-lysine-resistant AK and DHDPS variants on in vivo L-lysine production, *V. natriegens* strains expressing the native or engineered enzymes were constructed. The corresponding genes were cloned into a medium-copy plasmid under the control of an inducible P_tac_ promoter, and expression was induced with IPTG (1 mM) in early exponential phase. For L-lysine production, cell growth is typically restricted to redirect carbon flux from biomass formation to product synthesis. In industrial processes, this is commonly achieved through carbon-limited fed-batch cultivation (Cheng et al. 2018; Liu et al. 2022) or, alternatively in laboratory-scale studies, by growth-factor limitation in auxotrophic production strains (Kiss and Stephanopoulos 1991). Here, sulfur limitation was employed as a simple strategy to restrict growth in shake-flask cultures while maintaining excess carbon and nitrogen, without requiring additional strain engineering. Under these conditions, biomass formation ceases upon sulfur depletion, providing an initial screening platform for comparing the effects of different enzyme variants on L-lysine accumulation. Producer strains were cultivated in 25 mL of sulfur-limited VN mineral medium containing 20 g L^−1^ glucose and 150 mM NH_4_Cl, buffered with 200 mM MOPS. L-lysine production was quantified after 24 h of cell cultivation (Fig. 4, Online Resource Fig. S1).

L-lysine production by the control strain harboring an empty pMBI vector was negligible, reaching only 0.1 ± 0.0 mM. Likewise, individual (over)expression of AK variants resulted in no or only minor increases in L-lysine accumulation: 0.1 ± 0.0 mM (Vn.LysC1), 0.2 ± 0.1 mM (Vn.LysC1:T353I), and 0.4 ± 0.1 mM (Vn.LysC2). In contrast, individual overexpression of DHDPS variants yielded markedly higher L-lysine titers: wild-type *Vn.dapA1* overexpression produced 1.6 ± 0.1 mM L-lysine, while expression of the feedback-resistant *Vn.dapA1*:E84T mutant increased the titer to 6.0 ± 0.8 mM. Co-expression of the L-lysine-insensitive AK and DHDPS variants further affected L-lysine production: combination of the *Vn.lysC1*:T353I mutant with the *Vn.dapA1:E84T* mutant resulted in a pronounced reduction in L-lysine production (1.6 ± 0.1 mM) compared to expression of *Vn.dapA1*:E84T alone. In contrast, co-expression of the intrinsically L-lysine-resistant *Vn.lysC2* variant with the *Vn.dapA1*:E84T mutant yielded the highest L-lysine titers observed (9.0 ± 0.6 mM), with a corresponding yield of 0.11 ± 0.01 mol L-lysine per mol glucose. The strain produced L-lysine predominantly during the growth phase, before growth arrest induced by sulfur limitation (Online Resource Fig. S1), indicating that L-lysine formation was mainly growth-associated. Notably, glucose was not completely consumed during the 24-h cultivation period.

Because plasmid-based expression of AK variants had only a minor effect on L-lysine production, whereas expression of DHDPS variants yielded increased L-lysine titers, the contribution of the native chromosomal *lysC2* gene to L-lysine production was investigated further. To this end, L-lysine production was compared between wild-type and Δ*lysC2* strains expressing only *Vn.dapA1*:E84T from the plasmid. Comparable L-lysine titers were obtained in both strain backgrounds (Online Resource Fig. S3).

### Effects of additional pathway gene expression on L-lysine production

Having relieved feedback inhibition of AK and DHDPS, we next aimed to fully alleviate regulatory constraints along the L-lysine biosynthetic pathway. To this end, we targeted the *Vn.dapD* gene, which encodes tetrahydrodipicolinate succinylase (DapD) and is subject to transcriptional repression by L-lysine (Straube et al. 2026), for plasmid-based overexpression to circumvent this regulatory limitation. In parallel, we evaluated expression of a heterologous diaminopimelate dehydrogenase (DapDH) from *C. glutamicum* (*Cg.ddh*) as an alternative strategy: this enzyme catalyzes direct conversion of tetrahydrodipicolinate to meso-diaminopimelate, bypassing the native four-step conversion via* dapD* (Fig. 1).

**Fig. 5:**
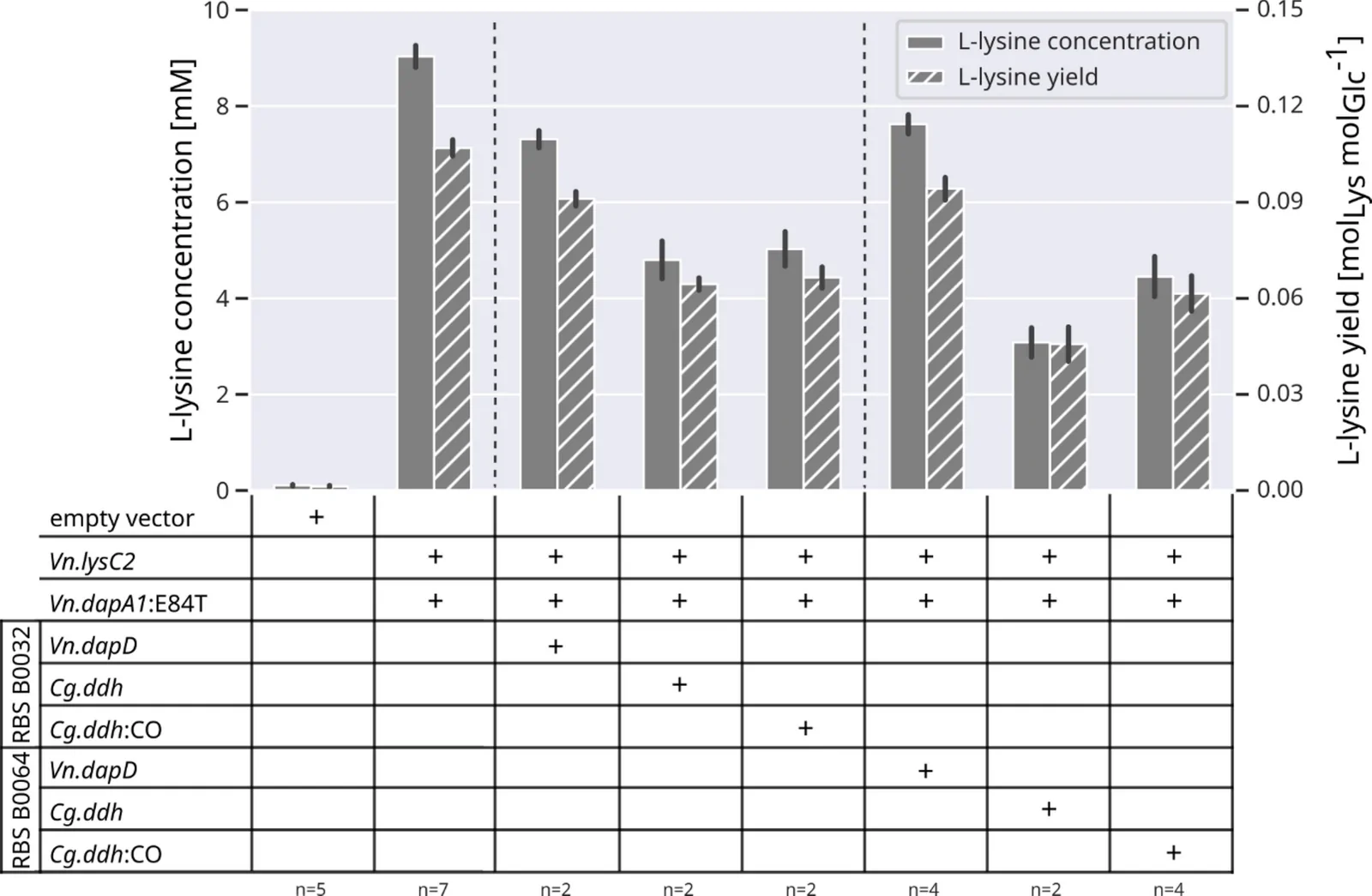
Effect of additional overexpression of native and heterologous pathway enzymes on L-lysine production. L-lysine concentration after 24 h and L-lysine yield are shown for *V. natriegens* DSM759 ∆*dns* strains harboring different pMBI vectors during growth on glucose. The table beneath the plot indicates, with “+,” the gene variant (and its RBS) expressed in each strain (25 mL S-limited VN medium with 20 g L^−1^ glucose and 150 mM NH_4_Cl; 1 mM IPTG; 37 °C; 220 rpm; Data are presented as mean ± SD; the number of biological replicates (n) is indicated for each condition)

To evaluate the impact of *Vn*.*dapD* or *Cg.ddh* (over)expression, the respective genes were introduced individually into the best-performing producer strain, which co-expresses feedback-resistant variants Vn.LysC2 and Vn.DapA1:E84T. Expression constructs were generated using one of two previously characterized RBSs differing in their reported translation strengths (B0032 and B0064) upstream of either *Vn*.*dapD* or *Cg.ddh*. For heterologous expression of *Cg.ddh*, both the wild-type gene and a manually codon-optimized variant (*Cg.ddh*:CO, Online Resource Text S1) were tested. Strains harboring the respective plasmids were cultivated in sulfur-limited VN mineral medium with 20 g L^−1^ glucose as the carbon source; gene expression was induced with 1 mM IPTG, and L-lysine accumulation was quantified after 24 h (Fig. 5, Online Resource Fig. S2).

Additional expression of either *Vn.dapD* or *Cg.ddh* did not further increase L-lysine production compared with the parent strain expressing only *Vn.lysC2* and *Vn.dapA1*:E84T. Using the medium-strength RBS (B0032), expression of *Vn.dapD* reduced the L-lysine titer to 81% of the parent strain, whereas expression of wild-type *Cg.ddh* and *Cg.ddh*:CO reduced the titer to 53% and 56%, respectively. Replacement of the medium-strength RBS with the high-strength variant (B0064) did not restore L-lysine production: L-lysine titers remained similar for *Vn.dapD* and *Cg.ddh*:CO, whereas expression of wild-type *Cg.ddh* further reduced the titer to 34% of the parent strain.

### L-lysine production using chitin monomers as carbon source

**Fig. 6:**
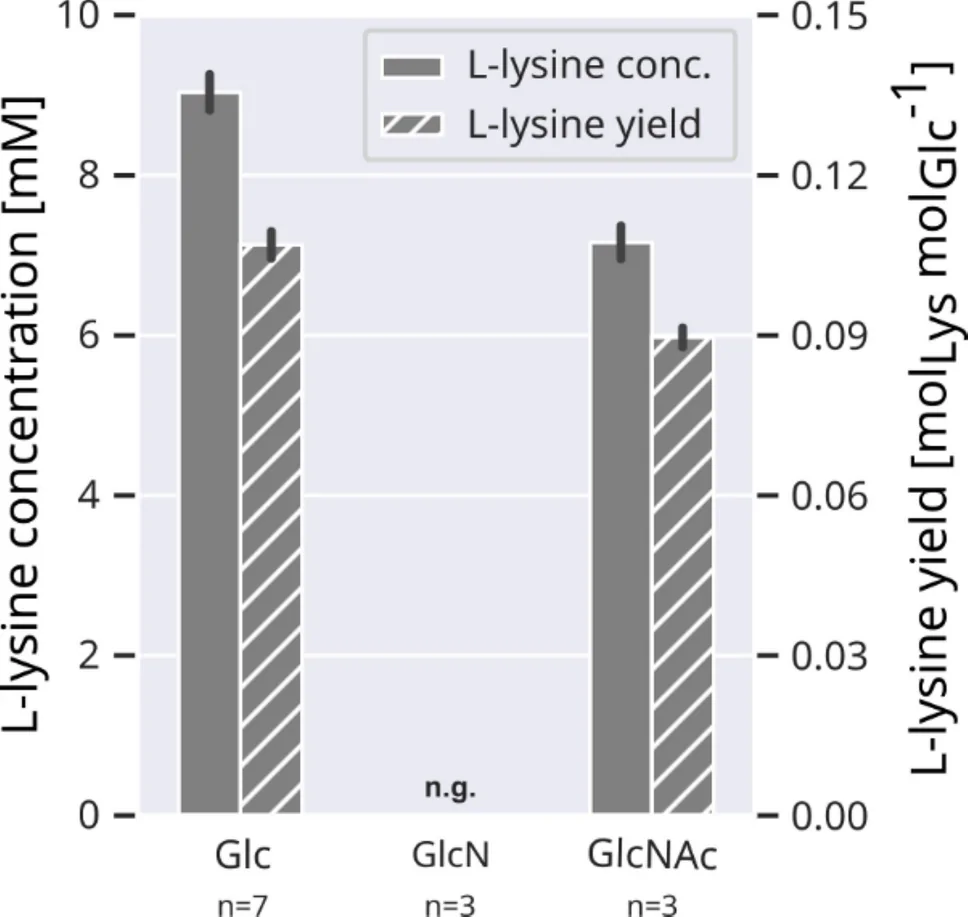
L-lysine production on chitin monomers as carbon source. L-lysine concentration after 24 h and L-lysine yield on respective carbon source are shown for *V. natriegens* DSM759 ∆*dns* harboring the pMBI_*Vn.lysC2*_*Vn.dapA1*:E84T vector (25-mL S-limited VN medium with 100-mM C-source and 150 mM NH_4_Cl; 1 mM IPTG; 37 °C; 220 rpm; n.g., no growth; data are presented as mean ± SD; the number of biological replicates (*n*) is indicated for each condition)

Chitin is the most abundant natural nitrogen-containing biopolymer and is widely found in crustacean shells, insect exoskeletons, and fungal cell walls. Large quantities of chitin-rich materials accumulate as low-value waste, particularly from seafood processing, making them an attractive and readily available feedstock for microbial production, especially of nitrogen-containing compounds such as amino acids. Because of its insolubility and structural complexity, microbial utilization of chitin typically relies on prior hydrolysis to the soluble monomers N-acetylglucosamine (GlcNAc) and glucosamine (GlcN) (Abidin et al. 2017; Ma et al. 2020). *V. natriegens* is particularly well suited for the utilization of these substrates, as it grows rapidly on both GlcN and GlcNAc (Hoffart et al. 2017). To evaluate L-lysine production from these chitin-derived substrates, the best-performing producer strain (co-expressing feedback-resistant Vn.LysC2 and Vn.DapA1:E84T) was cultivated in sulfur-limited VN mineral medium containing 100 mM GlcN or GlcNAc as the sole carbon source. L-lysine production was quantified after 24 h (Fig. 6, Online Resource Fig. S4).

Cultivation on GlcNAc yielded an L-lysine titer of 7.2 ± 0.29 mM and a yield of 0.09 ± 0.00 mol mol⁻^1^ after 24 h, comparable to those obtained with glucose as the carbon source. In contrast, no L-lysine production was detected during cultivation on GlcN because the producer strain failed to grow under these conditions. Because wild-type *V. natriegens* DSM759 is able to grow on GlcN in mineral medium (Hoffart et al. 2017), growth of plasmid-bearing strains on GlcN was investigated further. Both plasmid-bearing strains carrying either the empty control vector or the L-lysine production plasmid (pMBI_*Vn.lysC2*_*Vn.dapA1*:E84T) failed to grow in GlcN mineral medium. Supplementation with yeast extract under sulfur-limited conditions restored growth of the empty-vector control; however, the L-lysine-producing strain remained unable to grow under any of the tested conditions (Online Resource Fig. S5).

## Discussion

In this work, we constructed an L-lysine-producing *V. natriegens* DSM759 strain through a stepwise, rational engineering strategy targeting the native biosynthetic pathway. Building on our prior systems-level analysis of the strain’s genetic and regulatory architecture, we identified key metabolic bottlenecks and designed targeted interventions to relieve them.

### Overexpression of L-lysine-resistant AK and DHDPS enzymes leads to L-lysine production

The first engineering step addressed enzymatic regulation by relieving feedback inhibition of the key regulatory enzymes AK and DHDPS. This strategy represents a central principle of metabolic engineering for L-lysine production, as native L-lysine-sensitive enzymes support only limited L-lysine accumulation, whereas efficient production in established hosts such as *E. coli* and *C. glutamicum* generally requires feedback-resistant variants (Ohnishi et al. 2002; Becker et al. 2011; Chen et al. 2011a; Geng et al. 2013). Following this precedent, we engineered the native *V. natriegens* enzymes by introducing amino acid substitutions at conserved positions previously shown to confer feedback resistance in the homologous *E. coli* enzymes. For AK, four different Vn.LysC1 variants were generated. The substitutions affected basal enzyme activity in the absence of L-lysine, consistent with previous reports demonstrating that mutations within the regulatory domain can influence catalytic efficiency (Chen et al. 2011a, b; Xu et al. 2016a). All four variants exhibited reduced sensitivity to L-lysine compared to wild-type Vn.LysC1. Among the engineered variants, Vn.LysC1:T353I combined the greatest improvement in feedback resistance with increased catalytic activity and was therefore selected for subsequent in vivo evaluation in L-lysine production strains. As an alternative approach, the naturally L-lysine-insensitive isozyme Vn.LysC2 was likewise selected. Although its v_max_ was lower than that of wild-type Vn.LysC1 and Vn.LysC1:T353I, its intrinsic resistance to L-lysine inhibition made it an attractive native alternative to the engineered Vn.LysC1 variants. For DHDPS, engineering was limited to a single, well-characterized substitution. Accordingly, the conserved E84T mutation, previously shown to confer feedback resistance while preserving catalytic properties in the homologous Ec.DapA enzyme, was introduced into Vn.DapA1 (Geng et al. 2013). As observed for the *E. coli* homologue, the resulting Vn.DapA1:E84T variant exhibited reduced L-lysine sensitivity while retaining kinetic parameters comparable to those of the wild-type enzyme. Overall, the generation of feedback-resistant AK and DHDPS variants demonstrates that enzyme engineering strategies established for *E. coli* can be successfully transferred to the homologous native enzymes of *V. natriegens*.

Overexpression of the selected feedback-resistant AK and DHDPS variants was expected to relieve key regulatory bottlenecks in the native L-lysine biosynthetic pathway and thereby enable L-lysine production in *V. natriegens*. To evaluate their individual and combined contributions, the corresponding genes were expressed from plasmids in *V. natriegens* DSM759 Δ*dns* and L-lysine production was assessed in sulfur-limited shake-flask cultivations (Fig. 4). Expression of AK and DHDPS had distinct effects on L-lysine production. Whereas overexpression of AK variants alone resulted in no or only minor increases in L-lysine production, indicating that AK overexpression alone is insufficient to substantially increase flux towards L-lysine, expression of DHDPS variants yielded increased L-lysine titers, identifying DHDPS as the major bottleneck under the tested conditions. This is consistent with the central metabolic role of DHDPS, which catalyzes the first committed step towards L-lysine at the branch point where l-aspartate semialdehyde is also directed towards l-threonine, l-isoleucine, and l-methionine biosynthesis (Fig. 1). One possible explanation for the comparatively small contribution of AK overexpression was that flux through the AK node is not limiting under the tested conditions because it is sufficiently sustained by native expression of *Vn.lysC2*. However, deletion of the chromosomal *lysC2* gene did not reduce L-lysine production in strains expressing only *Vn.dapA1*:E84T, indicating that Vn.LysC2 is not essential for maintaining L-lysine production under these conditions. This result suggests that other native AK or AK-HD enzymes may contribute to sustaining flux through the AK node in *V. natriegens* (Straube et al. 2026), although the specific enzymes involved were not identified in the present study. In this context, Vn.LysC1 is unlikely to make a major contribution due to its strong sensitivity to L-lysine inhibition.

Combined expression of feedback-resistant AK and DHDPS variants revealed distinct effects depending on the AK enzyme used. Unexpectedly, co-expression of *Vn.lysC1*:T353I with *Vn.dapA1*:E84T reduced L-lysine production relative to expression of *Vn.dapA1*:E84T alone, despite the reduced L-lysine sensitivity of Vn.LysC1:T353I observed in vitro. Thus, the reduced feedback inhibition characterized in vitro did not result in increased L-lysine formation in vivo. One possible explanation is insufficient expression of *Vn.lysC1*:T353I, for example, due to sequence-dependent effects on translation arising from interactions between the upstream RBS and the coding sequence. However, because AK expression was not quantified, this remains a hypothesis that cannot be tested with the present data. Other factors, including intracellular substrate or cofactor availability and pathway-level flux constraints, may likewise contribute to the observed phenotype. In contrast, co-expression of the intrinsically L-lysine-insensitive Vn.LysC2 with Vn.DapA1:E84T yielded in the highest L-lysine titer (9.0 ± 0.6 mM) and yield (0.11 ± 0.01 mol_Lys_ mol_Glc_⁻^1^), indicating that expression of a feedback-insensitive AK can enhance L-lysine formation in vivo when combined with a feedback-resistant DHDPS. However, the differing performance of the AK variants was not fully predicted by their in vitro properties, indicating that catalytic characteristics measured under in vitro conditions do not necessarily translate directly to pathway performance in the cellular context. Notably, the observed L-lysine titer exceeded the sum of the individual contributions from *Vn.lysC2* and *Vn.dapA1*:E84T overexpression, consistent with a synergistic interaction between the two engineered pathway steps. Together, these findings demonstrate that targeted modification of key regulatory steps in the native L-lysine biosynthetic pathway can establish and enhance L-lysine formation in *V. natriegens*, in line with metabolic engineering strategies established for L-lysine production in *E. coli* and *C. glutamicum* (Becker et al. 2011; Geng et al. 2013). However, the present study does not establish why this strategy was effective for Vn.LysC2 but not Vn.LysC1:T353I, and therefore does not support generalization of the approach beyond the enzymes and conditions tested. Resolving whether the differential outcomes arise from differences in gene expression, intracellular metabolite availability, pathway flux distribution, downstream limitations, or other host-specific effects will require targeted mechanistic analyses. Despite this limitation, the strain co-expressing *Vn.lysC2* and *Vn.dapA1*:E84T achieved a yield of 0.11 ± 0.01 mol_Lys_ mol_Glc_⁻^1^ with only two targeted modifications, approaching the yield of 0.15 mol_Lys_ mol_Glc_⁻^1^ reported for a more extensively engineered *V. natriegens* L-lysine producer, which required extensive heterologous pathway expression and multiple chromosomal gene deletions (Nghiem et al. 2025). This yield nevertheless remains below those reported for more extensively engineered production systems, such as *C. glutamicum* strains carrying 12 genetic modifications, which reached a yield of 0.26 mol_Lys_ mol_Glc_⁻^1^ in shake-flask cultivation and 0.68 mol_Lys_ mol_Glc_⁻^1^ at bioreactor scale (Becker et al. 2011). These comparisons highlight the potential of minimal pathway engineering in *V. natriegens*, while also illustrating the substantial scope for further optimization.

### Overexpression of additional L-lysine pathway enzymes does not further increase product yield

To further increase pathway flux, we addressed transcriptional regulation through additional plasmid-based overexpression of native *Vn.dapD* or, alternatively, heterologous *Cg.ddh*. Neither intervention increased L-lysine production; instead, both strategies resulted in reduced titers and yields compared with the parent strain expressing only *Vn.lysC2* and *Vn.dapA1:E84T*. Although overexpression of *dapD* or *ddh* genes is an established strategy to improve L-lysine formation in *E. coli* and *C. glutamicum* (Becker et al. 2011; Xu et al. 2016a), our results indicate that amplification of this pathway segment is insufficient to increase L-lysine production in *V. natriegens* under the tested conditions. As expression levels of the target genes were not directly quantified, insufficient expression cannot be excluded as a possible explanation for the observed production decline. Other possible factors include suboptimal expression ratios among the pathway genes and the metabolic burden associated with plasmid-based overexpression. These possibilities suggest that further optimization of expression strength and balance, for example, through RBS libraries or chromosomal integration of engineered variants, could improve strain performance and stability. More broadly, the observed response may reflect constraints elsewhere in the pathway, in the supply of required precursors and cofactors, or in L-lysine export, which could represent additional engineering targets. In this context, increasing *dapD* or *ddh* expression without a corresponding increase in the capacity of other pathway steps may alter intermediate levels or pathway balance without increasing net L-lysine formation. In addition, the dehydrogenase route may have been limited by physiology: DapDH enzymes typically exhibit low affinity for ammonium, and elevated NH_4_^+^ concentrations are required to support L-lysine formation via this route (Xu et al. 2019; Liu et al. 2021). While overexpression of *Cg.ddh* has shown mixed effects on L-lysine formation in *E. coli*, an alternative DapDH from *Symbiobacterium thermophilum* has been reported to be more compatible and beneficial (Xu et al. 2016a, 2019). Thus, the present data do not allow the cause of the reduced production upon *dapD* or *ddh* overexpression to be distinguished among the proposed explanations, including insufficient or inappropriate expression, pathway imbalance, metabolic burden, and broader cofactor or physiological constraints. More generally, these findings also highlight the value of integrating stepwise pathway engineering with network-level analysis. While the stepwise approach used here provides a useful basis for isolating individual pathway contributions and generating mechanistic hypotheses, the effect of a given modification may depend on the expression balance of other pathway enzymes and their interaction with the surrounding metabolic network. Such context-dependent effects may not be readily identified when individual modifications are evaluated separately. Future optimization could therefore complement this approach with quantitative flux analysis and network-level modeling to determine where additional control can be exerted and to guide coordinated combinations of pathway modifications.

Alongside strain engineering, further development of the *V. natriegens* production platform will likely require process optimization. L-lysine formation occurred predominantly during growth before sulfur limitation and glucose remained incompletely consumed under the tested conditions, indicating scope to improve substrate utilization and extend the production phase. The sulfur-limited mineral medium used here provided a defined and reproducible basis for systematically evaluating the targeted enzyme variants and demonstrating the potential of the minimal engineering strategy under controlled conditions. Future process development should therefore focus on medium optimization and fed-batch cultivation, alongside assessment of key performance indicators such as volumetric productivity, specific production rate, and product yield. Together with further strain engineering, these approaches will be important to determine whether the rapid growth and high substrate uptake capacity of *V. natriegens* can be effectively leveraged to further improve L-lysine production towards levels relevant for industrial application.

### Chitin-derived N-acetylglucosamine supports L-lysine synthesis

The native ability of wild-type *V. natriegens* to utilize the chitin monomers GlcN and GlcNAc, together with its rapid growth and high substrate uptake rates (Hoffart et al. 2017), makes this host an interesting chassis for the conversion of chitin-derived substrates (Le et al. 2026). In the present study, L-lysine production from GlcNAc was achieved at levels comparable to those on glucose, demonstrating that GlcNAc can serve as a sole carbon and energy source for amino acid production in *V. natriegens*. Thus, the present results provide a basis for extending the developed production platform toward chitin-derived feedstocks. In contrast, no L-lysine formation was observed on GlcN, as the producer strain failed to grow under these cultivation conditions. This phenotype may reflect a combination of intrinsically less robust growth on GlcN and additional physiological burdens associated with the cultivation conditions and plasmid-bearing strains. In *E. coli*, growth on GlcN is slower than on GlcNAc due to differences in transport and regulatory induction of the shared GlcN/GlcNAc assimilation pathway (Fig. 1) (Álvarez-Añorve et al. 2005, 2009, 2016). Wild-type *V. natriegens* exhibits a similar growth pattern (Hoffart et al. 2017), suggesting that comparable limitations may affect GlcN utilization in this organism. We therefore hypothesize that GlcN-supported growth tolerates only a limited additional physiological load, which is progressively consumed by burdens such as growth in defined medium, sulfur limitation, plasmid maintenance, and additional gene expression, until growth is no longer sustained. Consistent with this interpretation, plasmid-bearing strains failed to grow on GlcN, although growth of the empty-vector control could be restored by yeast extract supplementation under sulfur-limited conditions (Online Resource Fig. S5). Notably, however, the producer strain did not resume growth under these conditions. Since the production genes are expressed from the same plasmid, the present data cannot distinguish whether this defect is caused by flux into the L-lysine pathway, by the burden of expressing two additional genes, or by a combination of both. Comparative metabolomic or other systems-level analyses, comparing the producer strain and the empty-vector control under GlcN cultivation, could therefore help to identify the underlying metabolic perturbations and clarify the cause of this growth defect. These findings nevertheless indicate that L-lysine production from GlcN will likely require targeted engineering of chitin monomer uptake and assimilation pathways. This is particularly relevant because chemical hydrolysis of chitin frequently produces mixtures of GlcNAc and GlcN as a result of deacetylation (Einbu and Vårum 2008). In addition to strain engineering, co-feeding strategies, such as adding a small amount of GlcNAc to GlcN, may help induce the pathway and improve GlcN utilization (Le et al. 2026).

## Conclusion

In summary, we constructed a minimally engineered yet efficient L-lysine-producing *V. natriegens* DSM759 strain through rational, stepwise optimization of its native biosynthetic pathway. Targeted relief of feedback inhibition of the key regulatory enzymes AK and DHDPS was sufficient to establish L-lysine production without extensive genetic modifications, demonstrating the potential of native pathway engineering in this emerging production host. Furthermore, the engineered strain produced L-lysine from the chitin-derived monomer GlcNAc at levels comparable to those obtained with glucose, supporting the use of GlcNAc as a substrate for L-lysine production in *V. natriegens*. In contrast, the strain’s inability to utilize GlcN highlights the potential for metabolic engineering to broaden the range of chitin-derived substrates that can be converted into value-added products. Together, these findings provide a foundation for further metabolic and process engineering to advance L-lysine production in *V. natriegens* and explore its potential for the valorization of chitin-derived feedstocks in a sustainable biorefinery framework.

## Supplementary information

Below is the link to the electronic supplementary material.

Supplementary file1 (PDF 801 kb)

## Data Availability

Data will be made available on request.

## References

[CR1] Abidin MZ, Junqueira-Gonçalves MP, Khutoryanskiy VV, Niranjan K (2017) Intensifying chitin hydrolysis by adjunct treatments – an overview. J Chem Technol Biotechnol 92:2787–2798. 10.1002/jctb.5208

[CR2] Álvarez-Añorve LI, Calcagno ML, Plumbridge J (2005) Why does *Escherichia coli* grow more slowly on glucosamine than on N-acetylglucosamine? Effects of enzyme levels and allosteric activation of GlcN6P deaminase (NagB) on growth rates. J Bacteriol 187:2974–2982. 10.1128/jb.187.9.2974-2982.2005 PMC1082822 15838023

[CR3] Álvarez-Añorve LI, Bustos-Jaimes I, Calcagno ML, Plumbridge J (2009) Allosteric regulation of glucosamine-6-phosphate deaminase (NagB) and growth of *Escherichia coli* on glucosamine. J Bacteriol 191:6401–6407. 10.1128/jb.00633-09 PMC2753035 19700525

[CR4] Álvarez-Añorve LI, Gaugué I, Link H, Marcos-Viquez J, Díaz-Jiménez DM, Zonszein S, Bustos-Jaimes I, Schmitz-Afonso I, Calcagno ML, Plumbridge J (2016) Allosteric activation of *Escherichia coli* glucosamine-6-phosphate deaminase (NagB) in vivo justified by intracellular amino sugar metabolite concentrations. J Bacteriol 198:1610–1620. 10.1128/jb.00870-15 PMC4959280 27002132

[CR5] Becker J, Zelder O, Häfner S, Schröder H, Wittmann C (2011) From zero to hero—design-based systems metabolic engineering of *Corynebacterium glutamicum* for L-lysine production. Metab Eng 13:159–168. 10.1016/j.ymben.2011.01.003 21241816

[CR6] Chen Z, Meyer W, Rappert S, Sun J, Zeng A-P (2011) Coevolutionary analysis enabled rational deregulation of allosteric enzyme inhibition in *Corynebacterium glutamicum* for lysine production. Appl Environ Microbiol 77:4352–4360. 10.1128/AEM.02912-10 PMC3127711 21531824

[CR7] Chen Z, Rappert S, Sun J, Zeng A-P (2011) Integrating molecular dynamics and co-evolutionary analysis for reliable target prediction and deregulation of the allosteric inhibition of aspartokinase for amino acid production. J Biotechnol 154:248–254. 10.1016/j.jbiotec.2011.05.005 21609739

[CR8] Cheng J, Chen P, Song A, Wang D, Wang Q (2018) Expanding lysine industry: industrial biomanufacturing of lysine and its derivatives. J Ind Microbiol Biotechnol 45:719–734. 10.1007/s10295-018-2030-8 29654382

[CR9] Dalia TN, Hayes CA, Stolyar S, Marx CJ, McKinlay JB, Dalia AB (2017) Multiplex genome editing by natural transformation (MuGENT) for synthetic biology in *Vibrio natriegens*. ACS Synth Biol 6:1650–1655. 10.1021/acssynbio.7b00116 PMC6519440 28571309

[CR10] Einbu A, Vårum KM (2008) Characterization of chitin and its hydrolysis to GlcNAc and GlcN. Biomacromol 9:1870–1875. 10.1021/bm8001123 18540645

[CR11] Ellis GA, Tschirhart T, Spangler J, Walper SA, Medintz IL, Vora GJ (2019) Exploiting the feedstock flexibility of the emergent synthetic biology chassis *Vibrio natriegens* for engineered natural product production. Mar Drugs 17. 10.3390/md17120679 PMC6950413 31801279

[CR12] Erian AM, Freitag P, Gibisch M, Pflügl S (2020) High rate 2,3-butanediol production with *Vibrio natriegens*. Bioresour Technol Rep 10. 10.1016/j.biteb.2020.100408

[CR13] Faber A, Politan RJ, Stukenberg D, Morris KM, Kim R, Jeon E, Inckemann R, Becker A, Thuronyi B, Fritz G (2025) Expanding genetic engineering capabilities in *Vibrio natriegens* with the Vnat collection. Nucleic Acids Res 53. 10.1093/nar/gkaf580 PMC12255299 40650977

[CR14] Faber A, Politan R, Nicol A, Della Valle S, Jenkins B, Flematti G, Fritz G (2026) Mitigating protocatechuic acid toxicity in *Vibrio natriegens* enables poly-3-hydroxybutyrate production from an aromatic carbon source. Appl Microbiol Biotechnol 110:179. 10.1007/s00253-026-13831-z PMC13253662 42045670

[CR15] Geng F, Chen Z, Zheng P, Sun J, Zeng A-P (2013) Exploring the allosteric mechanism of dihydrodipicolinate synthase by reverse engineering of the allosteric inhibitor binding sites and its application for lysine production. Appl Microbiol Biotechnol 97:1963–1971. 10.1007/s00253-012-4062-8 22644522

[CR16] Gruber AR, Lorenz R, Bernhart SH, Neuböck R, Hofacker IL (2008) The Vienna RNA websuite. Nucleic Acids Res 36:W70–W74. 10.1093/nar/gkn188 PMC2447809 18424795

[CR17] Hädrich M, Schulze C, Hoff J, Blombach B (2025) Vibrio natriegens: application of a fast-growing halophilic bacterium. In: Holtmann D (ed) Unconventional organisms in biotechnology. Springer Nature Switzerland, Cham, pp 85–116 10.1007/10_2024_271 39527262

[CR18] Hoff J, Daniel B, Stukenberg D, Thuronyi BW, Waldminghaus T, Fritz G (2020) *Vibrio natriegens*: an ultrafast-growing marine bacterium as emerging synthetic biology chassis. Environ Microbiol 22:4394–4408. 10.1111/1462-2920.15128 32537803

[CR19] Hoffart E, Grenz S, Lange J, Nitschel R, Müller F, Schwentner A, Feith A, Lenfers-Lücker M, Takors R, Blombach B (2017) High substrate uptake rates empower *Vibrio natriegens* as production host for industrial biotechnology. Appl Environ Microbiol 83:e01614-e1617. 10.1128/AEM.01614-17 PMC5666143 28887417

[CR20] Holeček M (2025) Lysine: sources, metabolism, physiological importance, and use as a supplement. Int J Mol Sci 26. 10.3390/ijms26188791 PMC12469518 41009362

[CR21] Jorge JMP, Pérez-García F, Wendisch VF (2017) A new metabolic route for the fermentative production of 5-aminovalerate from glucose and alternative carbon sources. Bioresour Technol 245:1701–1709. 10.1016/j.biortech.2017.04.108 28522202

[CR22] Kanehisa M, Goto S (2000) KEGG: Kyoto Encyclopedia of Genes and Genomes. Nucleic Acids Res 28:27–30. 10.1093/nar/28.1.27 PMC102409 10592173

[CR23] Kikuchi Y, Kojima H, Tanaka T (1999) Mutational analysis of the feedback sites of lysine-sensitive aspartokinase of *Escherichia coli*. FEMS Microbiol Lett 173:211–215. 10.1111/j.1574-6968.1999.tb13504.x 10220897

[CR24] Kiss RD, Stephanopoulos G (1991) Metabolic activity control of the L-lysine fermentation by restrained growth fed-batch strategies. Biotechnol Prog 7:501–509. 10.1021/bp00012a004

[CR25] Kotaka M, Ren J, Lockyer M, Hawkins AR, Stammers DK (2006) Structures of R- and T-state *Escherichia coli* aspartokinase III. J Biol Chem 281:31544–31552. 10.1016/S0021-9258(19)84068-1 16905770

[CR26] Kumar R, Shah S, Paramita Das P, Bhagavanbhai GGK, Al Fatesh A, Chowdhury B (2019) An overview of caprolactam synthesis. Catal Rev 61:516–594. 10.1080/01614940.2019.1650876

[CR27] Le T, Nguyen T-H, Vu D-C, Cao T-H-T, Vu OT-K, Nguyen T-T, Pham T-A, Le T-H (2026) High-cell-density cultivation of *Vibrio natriegens* N5.3 on chitin monomers: a step toward chitin valorization. Biotechnol Lett 48. 10.1007/s10529-026-03696-7 41575616

[CR28] Lee HH, Ostrov N, Wong BG, Gold MA, Khalil AS, Church GM (2019) Functional genomics of the rapidly replicating bacterium *Vibrio natriegens* by CRISPRi. Nat Microbiol 4:1105–1113. 10.1038/s41564-019-0423-8 30962569

[CR29] Li S, Mao Y, Zhang L, Wang M, Meng J, Liu X, Bai Y, Guo Y (2022) Recent advances in microbial ε-poly-L-lysine fermentation and its diverse applications. Biotechnol Biofuels Bioprod 15. 10.1186/s13068-022-02166-2 PMC9205021 35710433

[CR30] Liu N, Zhang T-T, Rao Z-M, Zhang W-G, Xu J-Z (2021) Reconstruction of the diaminopimelic acid pathway to promote L-lysine production in *Corynebacterium glutamicum*. Int J Mol Sci 22. 10.3390/ijms22169065 PMC8396482 34445771

[CR31] Liu J, Xu J-Z, Rao Z-M, Zhang W-G (2022) Industrial production of L-lysine in *Corynebacterium glutamicum*: progress and prospects. Microbiol Res 262. 10.1016/j.micres.2022.127101 35803058

[CR32] Lopez MJ, Mohiuddin SS (2025) Biochemistry, essential amino acids. StatPearls Publishing, Treasure Island (FL) 32496725

[CR33] Ma X, Gözaydın G, Yang H, Ning W, Han X, Poon NY, Liang H, Yan N, Zhou K (2020) Upcycling chitin-containing waste into organonitrogen chemicals via an integrated process. Proc Natl Acad Sci U S A 117:7719–7728. 10.1073/pnas.1919862117 PMC7149430 32213582

[CR34] Madeira F, Madhusoodanan N, Lee J, Eusebi A, Niewielska A, Tivey ARN, Lopez R, Butcher S (2024) The EMBL-EBI Job Dispatcher sequence analysis tools framework in 2024. Nucleic Acids Res 52:W521–W525. 10.1093/nar/gkae241 PMC11223882 38597606

[CR35] Nghiem TT, Tran HL, Nguyen TT, Le TH, Le QH (2025) Unlocking the potential of *Vibrio natriegens* for L-lysine production. Res J Biotechnol 11:1. 10.25303/2011rjbt0109

[CR36] Ogawa-Miyata Y, Kojima H, Sano K (2001) Mutation analysis of the feedback inhibition site of aspartokinase III of *Escherichia coli* K-12 and its use in L-threonine production. Biosci Biotechnol Biochem 65:1149–1154. 10.1271/bbb.65.1149 11440130

[CR37] Ohnishi J, Mitsuhashi S, Hayashi M, Ando S, Yokoi H, Ochiai K, Ikeda M (2002) A novel methodology employing *Corynebacterium glutamicum* genome information to generate a new L-lysine-producing mutant. Appl Microbiol Biotechnol 58:217–223. 10.1007/s00253-001-0883-6 11876415

[CR38] Politan RJ, Della Valle S, Pineda L, Joshi J, Euler C, Flematti G, Fritz G (2025) Establishing *Vibrio natriegens* as a high-performance host for acetate-based poly-3-hydroxybutyrate production. Metab Eng 92:22–38. 10.1016/j.ymben.2025.07.003 40653003

[CR39] Stella RG, Baumann P, Lorke S, Münstermann F, Wirtz A, Wiechert J, Marienhagen J, Frunzke J (2021) Biosensor-based isolation of amino acid-producing *Vibrio natriegens* strains. Metab Eng Commun 13. 10.1016/j.mec.2021.e00187 PMC8605253 34824977

[CR40] Straube E, Radde J, Tran TVA, Yazdi NK, Blanco RC, Le HT, Frazão CJR, Walther T (2026) Genetic make-up and regulation of the L-lysine biosynthesis pathway in *Vibrio natriegens*. Microb Cell 13:44–62. 10.15698/mic2026.02.867 PMC12925634 41732680

[CR41] Stukenberg D, Hensel T, Hoff J, Daniel B, Inckemann R, Tedeschi JN, Nousch F, Fritz G (2021) The Marburg collection: a golden gate DNA assembly framework for synthetic biology applications in *Vibrio natriegens*. ACS Synth Biol 10:1904–1919. 10.1021/acssynbio.1c00126 34255476

[CR42] Stukenberg D, Hoff J, Faber A, Becker A (2022) NT-CRISPR, combining natural transformation and CRISPR-Cas9 counterselection for markerless and scarless genome editing in *Vibrio natriegens*. Commun Biol 5:1–13. 10.1038/s42003-022-03150-0 PMC8956659 35338236

[CR43] Thoma F, Blombach B (2021) Metabolic engineering of *Vibrio natriegens*. Essays Biochem 65:381–392. 10.1042/EBC20200135 PMC8314017 33835156

[CR44] Thoma F, Schulze C, Gutierrez-Coto C, Hädrich M, Huber J, Gunkel C, Thoma R, Blombach B (2022) Metabolic engineering of *Vibrio natriegens* for anaerobic succinate production. Microb Biotechnol 15:1671–1684. 10.1111/1751-7915.13983 PMC9151343 34843164

[CR45] Wang Y, Li Q, Zheng P, Guo Y, Wang L, Zhang T, Sun J, Ma Y (2016) Evolving the L-lysine high-producing strain of *Escherichia coli* using a newly developed high-throughput screening method. J Ind Microbiol Biotechnol 43:1227–1235. 10.1007/s10295-016-1803-1 PMC4983297 27369765

[CR46] Wang Z, Tschirhart T, Schultzhaus Z, Kelly EE, Chen A, Oh E, Nag O, Glaser ER, Kim E, Lloyd PF, Charles PT, Li W, Leary D, Compton J, Phillips DA, Dhinojwala A, Payne GF, Vora GJ (2020) Melanin produced by the fast-growing marine bacterium *Vibrio natriegens* through heterologous biosynthesis: characterization and application. Appl Environ Microbiol 86. 10.1128/AEM.02749-19 PMC7028964 31836580

[CR47] Weinstock MT, Hesek ED, Wilson CM, Gibson DG (2016) *Vibrio natriegens* as a fast-growing host for molecular biology. Nat Methods 13:849–851. 10.1038/nmeth.3970 27571549

[CR48] Wu F, Wang S, Peng Y, Guo Y, Wang Q (2023) Metabolic engineering of fast-growing *Vibrio natriegens* for efficient pyruvate production. Microb Cell Fact 22:172. 10.1186/s12934-023-02185-0 PMC10476420 37667234

[CR49] Wu Z, Chen T, Sun W, Chen Y, Ying H (2024) Optimizing *Escherichia coli* strains and fermentation processes for enhanced L-lysine production: a review. Front Microbiol. 10.3389/fmicb.2024.1485624 PMC11486702 39430105

[CR50] Xu J, Han M, Ren X, Zhang W (2016) Modification of aspartokinase III and dihydrodipicolinate synthetase increases the production of L-lysine in *Escherichia coli*. Biochem Eng J 114:79–86. 10.1016/j.bej.2016.06.025

[CR51] Xu Z, Xu Z, Feng X, Xu D, Liang J, Xu H (2016) Recent advances in the biotechnological production of microbial poly(ɛ-L-lysine) and understanding of its biosynthetic mechanism. Appl Microbiol Biotechnol 100:6619–6630. 10.1007/s00253-016-7677-3 27333910

[CR52] Xu J-Z, Ruan H-Z, Liu L-M, Wang L-P, Zhang W-G (2019) Overexpression of thermostable meso-diaminopimelate dehydrogenase to redirect diaminopimelate pathway for increasing L-lysine production in *Escherichia coli*. Sci Rep 9. 10.1038/s41598-018-37974-w PMC6382763 30787467

[CR53] Ye C, Luo Q, Guo L, Gao C, Xu N, Zhang L, Liu L, Chen X (2020) Improving lysine production through construction of an *Escherichia coli* enzyme-constrained model. Biotechnol Bioeng 117:3533–3544. 10.1002/bit.27485 32648933

[CR54] Yin L, Zhou Y, Ding N, Fang Y (2024) Recent advances in metabolic engineering for the biosynthesis of phosphoenol pyruvate–oxaloacetate–pyruvate-derived amino acids. Molecules 29:2893. 10.3390/molecules29122893 PMC11206799 38930958

[CR55] Ying H, He X, Li Y, Chen K, Ouyang P (2014) Optimization of culture conditions for enhanced lysine production using engineered *Escherichia coli*. Appl Biochem Biotechnol 172:3835–3843. 10.1007/s12010-014-0820-7 24682878

[CR56] Zhang Y, Li Z, Liu Y, Cen X, Liu D, Chen Z (2021) Systems metabolic engineering of *Vibrio natriegens* for the production of 1,3-propanediol. Metab Eng 65:52–65. 10.1016/j.ymben.2021.03.008 33722653

[CR57] Zheng L, Baumann U, Reymond J-L (2004) An efficient one-step site-directed and site-saturation mutagenesis protocol. Nucleic Acids Res 32. 10.1093/nar/gnh110 PMC514394 15304544

