# Supplementary material for: L-Lysine production from glucose and chitin monomers using engineered Vibrio natriegens
Source: Appl Microbiol Biotechnol. 2026 Nov 4;110:335. doi: 10.1007/s00253-026-14090-8 (PMC13737242; doi:10.1007/s00253-026-14090-8)
Supplement: Supplementary file 1 [file 253_2026_14090_MOESM1_ESM.pdf]

Applied Microbiology and Biotechnology

Supplementary Data

L-Lysine production from glucose and chitin monomers using engineered *Vibrio natriegens*

Elly Straube[1], Thi Van Anh Tran[1], Anna Faber[234], Nadine Ihle[1], Rubén Crespo Blanco[1], Ha Thanh Le[5], Georg Fritz[24], Cláudio J. R. Frazão[1], Thomas Walther[1*]

Author affiliations:

[1] Institute of Natural Materials Technology, TU Dresden, 01062 Dresden, Germany

[2] School of Molecular Sciences, The University of Western Australia, Crawley 6009, Australia

[3] Forrest Research Foundation, Crawley 6009, Australia

[4] Oceans Institute, The University of Western Australia, Crawley 6009, Australia

[5] School of Chemistry and Life Science, Hanoi University of Science and Technology, 1 Dai Co Viet, 9 Hanoi, Vietnam

*** Correspondence:**

Thomas Walther

thomas_walther@tu-dresden.de

Text S1| Codon-optimised *Cg.ddh* derivative. Manually optimized codons are highlighted in red.

atgaccaacatccgtgtagctatcgtgggctacggaaacctgggacgcagcgtcgaaaagcttattgccaagcagccagacatggaccttgtag
gaatcttctcgcgccgtgccaccctcgacacaaagacgccagtctttgatgtcgccgacgtggacaagcacgccgacgacgtggacgtgctgttc
ctgtgcatgggctccgccaccgacatccctgagcaggcaccaaagttcgcgcagttcgcctgcaccgtagacacctacgacaaccaccgcgacat
cccacgccaccgccaggtcatgaacgaagccgccaccgcagccggcaacgttgcactggtctctaccggctgggatccaggaatgttctccatca
accgcgtctacgcagcggcagtcttagccgagcaccagcagcacaccttctggggcccaggtttgtcacagggccactccgatgctttgcgacgc
atccctggcgttcaaaaggcagtccagtacaccctcccatccgaagacgccctggaaaaggcccgccgcggcgaagccggcgaccttaccgga
aagcaaacccacaagcgccaatgcttcgtggttgccgacgcggccgatcacgagcgcatcgaaaacgacatccgcaccatgcctgattacttcgt
tggctacgaagtcgaagtcaacttcatcgacgaagcaaccttcgactccgagcacaccggcatgccacacggtggccacgtgattaccaccggc
gacaccggtggcttcaaccacaccgtggaatacatcctcaagctggaccgaaacccagatttcaccgcttcctcacagatcgctttcggtcgcgca
gctcaccgcatgaagcagcagggccaaagcggagctttcaccgtcctcgaagttgctccatacctgctctccccagagaacttggacgatctgat
cgcacgcgacgtctaa

Table S1| Primers used in this study.

Primer	Sequence (5'→3')
Primers for site-directed mutagenesis (SDM)	
SDM-*Vn.lysC1*:E251K-fw	gaagcgtcaAAAatggcaaacttcggtgc
SDM-*Vn.lysC1*:E251K-rv	gcaccgaagtttgccatTTTgacgcttc
SDM-*Vn.lysC1*:V340A-fw	GCAgacctgattaccacttcagaaat
SDM-*Vn.lysC1*:V340A-rv	TGCtgaaattttgtgtttagccagaatctcg
SDM-*Vn.lysC1*:D341P-fw	CCActgattaccacttcagaaatcag
SDM-*Vn.lysC1*:D341P-rv	TGGcactgaaattttgtgtttagcca
SDM-*Vn.lysC1*:T353I-fw	gtttcgttaATCctagaccaaacagac
SDM-*Vn.lysC1*:T353I-rv	tttggtctagGATtaacgaaacactgatttc
SDM-*Vn.dapA1*:E84T-fw	cgggtgcaaatgctacccacACCgccgtgacgttcag
SDM-*Vn.dapA1*:E84T-rv	agtttactgaacgtcacggcGGTgtgggtagcatttgcac
Construction of pMBI vectors via isothermal assembly (IA) or restriction-ligation-based cloning (RL)	
IA-bb-fw	agtcacacaggaaagtaatcgcttaaccaggcatcaaataaaac
IA-bb-rv	gattactttcctgtgtgactc
IA-*Vn.lysC1*(bb)-fw	agtcacacaggaaagtaatcaATGAGCGCATTTAACGTAG
IA-*Vn.lysC1*-rv	TTATTTTTCAAATAGCTCAGC
IA-*Vn.lysC1*(bb)-rv	gttttatttgatgcctggttATTTTTCAAATAGCTCAGCATG
IA-*Vn.lysC2*(bb)-fw	agtcacacaggaaagtaatcaATGAAAAAGCCCCTTATCG
IA-*Vn.lysC2*-rv	TCACCCTATTAGGGCATG
IA-*Vn.lysC2*(bb)-rv	gttttatttgatgcctggttACCCTATTAGGGCATGTTTTTG
IA-*Vn.dapA1*(bb)-fw	agtcacacaggaaagtaatcaATGTTTTCAGGAAGTATCG
IA-*Vn.dapA1*(*Vn.lysC1*)-fw	ctgagctatttgaaaaataatactagagtcacacaggactaatcaATGTTTTCAGGAAGTATCG
IA-*Vn.dapA1*(*Vn.lysC2*)-fw	aacatgccctaatagggtgatactagagtcacacaggactaatcaATGTTTTCAGGAAGTATCG
IA-*Vn.dapA1*-rv	TTAATCTTTATAAATACGGGCG
IA-*Vn.dapA1*(bb)-rv	gttttatttgatgcctggttAATCTTTATAAATACGGGCG
IA-*Vn.dapD*-fw	cccgtatttataaagattaatactagagaaagaggggaaataatcaATGGCTTTCTTTTCTCTAG
IA-*Vn.dapD*-rv	gttttatttgatgcctggttAGTTGTTTGAGTGTAGTTC
RL-bb-fw	attcgtgtcgaCCAGGCATCAAATAAAACG
RL-bb-rv	tagactcatatgttaatctttataaatacgggcgtc
RL-*Cg.ddh*-fw	attcgtcatatgTACTAGAGAAAGAGGGGAAATAATCAatgaccaacatccgcgtagc
RL-*Cg.ddh*:CO-fw	attcgtcatatgTACTAGAGAAAGAGGGGAAATAATCAatgaccaacatccgtgtagc
RL-*Cg.ddh*-rv	tagactgtcgacttagacgtcgcgtgcgatc
Plasmid verification primers	
ver-pET28-fw	ATGCGTCCGGCGTAGA
ver-pET28-rv	CTAGTTATTGCTCAGCGGT
ver-pMBI-fw	CGGGAAACGGTCATATAAG
ver-pMBI-rv	CTCTAGTAGAGAGCGTTCAC

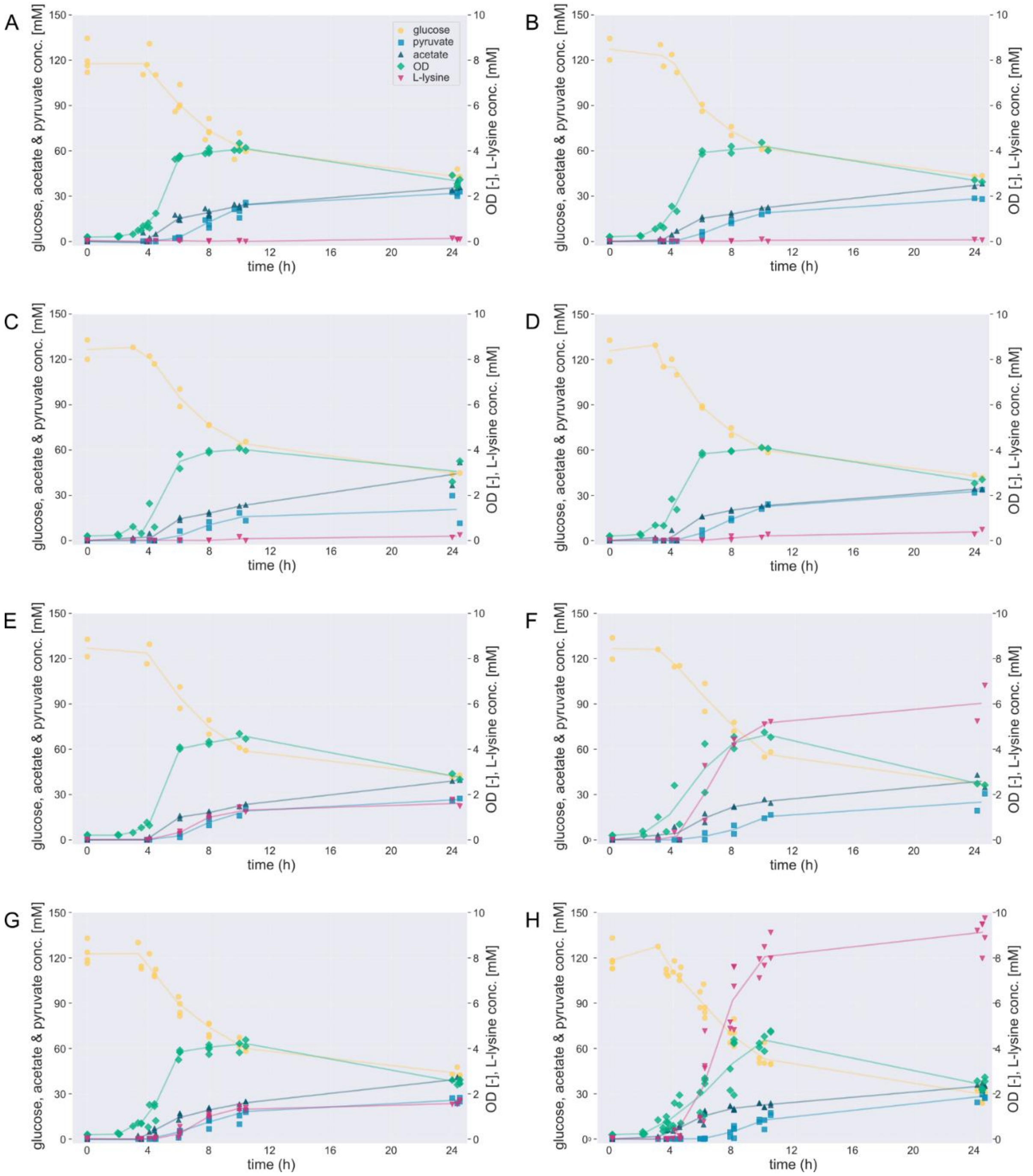

Fig. S1: Cultivation profiles of *V. natriegens* Δ*dns* harboring (A) the pMBI empty plasmid, (B) the pMBI_*Vn.lysC1*, (C) the pMBI_*Vn.lysC1*:T353I, (D) the pMBI_*Vn.lysC2*, (E) the pMBI_*Vn.dapA1*, (F) the pMBI_*Vn.dapA1*:E84T, (G) the pMBI_*Vn.lysC1*:T353I_*Vn.dapA1*:E84T and (H) the pMBI_*Vn.lysC2*_Vn.*dapA1*:E84T vector during growth on glucose. Time-course data showing biomass accumulation ($OD_{600\ nm}$), glucose consumption, and formation of pyruvate, acetate, and L-lysine. Scatter points show individual raw measurements. Lines represent tolerance-based mean values, where measurements within 0.5 h were grouped and averaged to visualize the central trend without altering the original data (25 mL S-limited VN medium with 120 mM glucose and 150 mM NH_4Cl, 1 mM IPTG, 37°C, 220 rpm, n≥2).

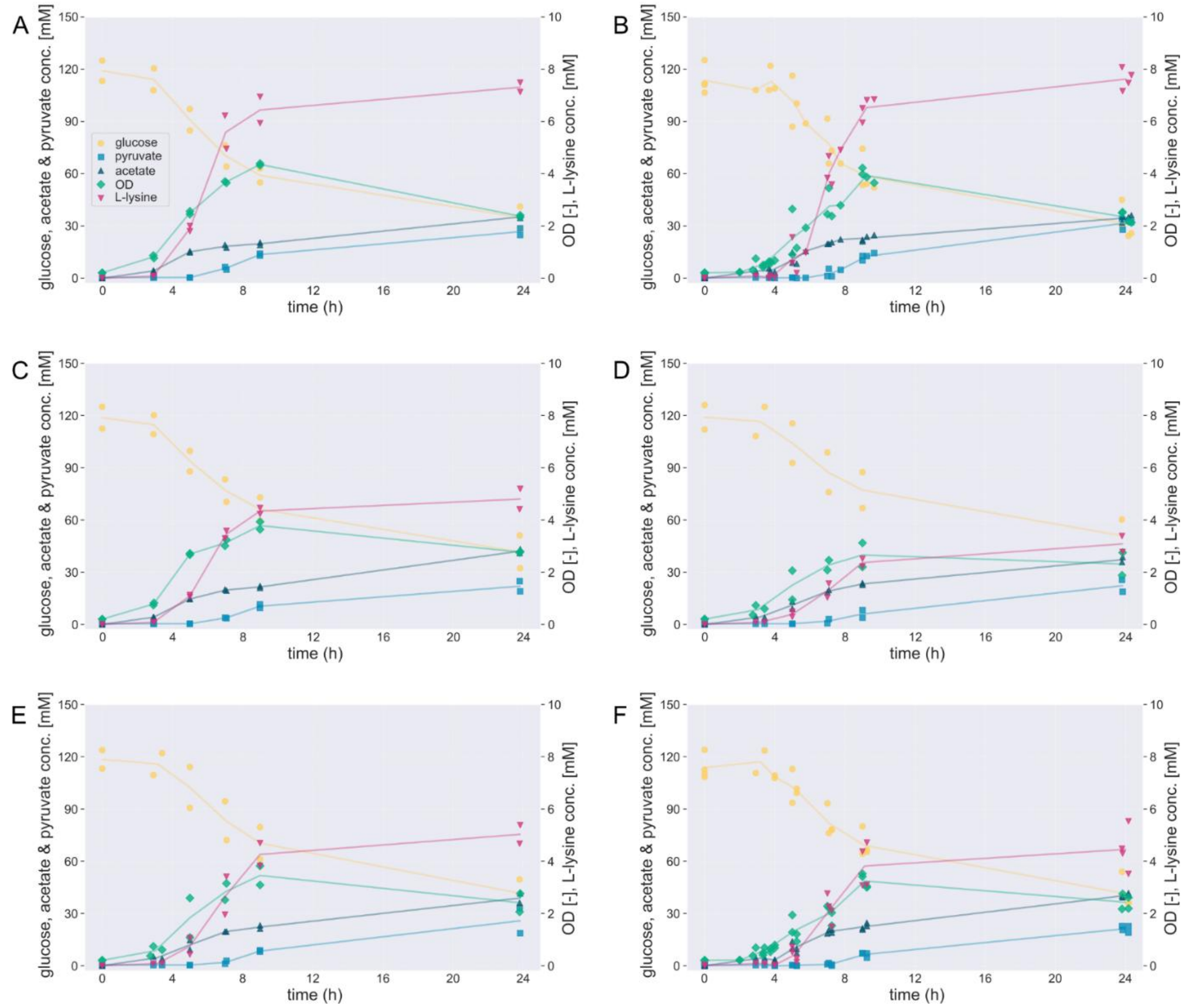

Fig. S2: Cultivation profiles of six *V. natriegens* Δ*dns* strains during growth on glucose (A-F), each overexpressing additional L-lysine pathway enzymes from pMBI vectors. All plasmids share the same two upstream genes *(Vn.lysC2* and *Vn.dapA1*:E84T*)* , while the third gene and its RBS vary: (A) *Vn.dapD*-B0032, (B) *Vn.dapD*-B0064, (C) *Cg.ddh*-B0032, (D) *Cg.ddh*-B0064, (E) *Cg.ddh:*CO-B0032, and (F) *Cg.ddh:*CO-B0064. Time-course data showing biomass accumulation ($OD_{600\ nm}$), glucose consumption, and formation of pyruvate, acetate, and L-lysine. Scatter points show individual raw measurements. Lines represent tolerance-based mean values, where measurements within 0.5 h were grouped and averaged to visualize the central trend without altering the original data (25 mL S-limited VN medium with 120 mM glucose and 150 mM NH_4Cl, 1 mM IPTG, 37°C, 220 rpm, n≥2).

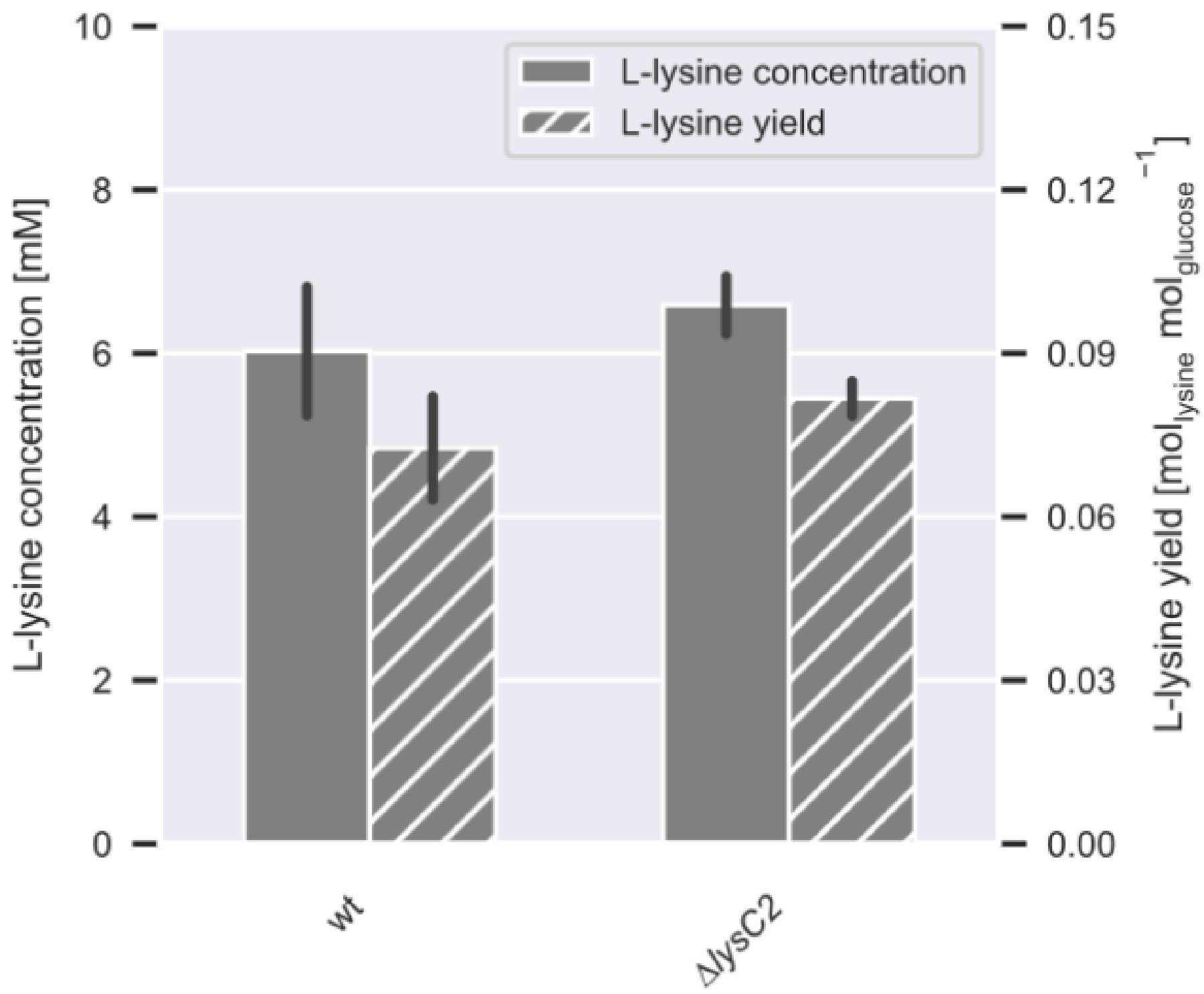

Fig. S3: Effect of *lysC2* deletion on L-lysine production in *V. natriegens* strains expressing the L-lysine insensitive *Vn.dapA1*:E84T variant. L-lysine concentration after 24 h of cultivation and L-lysine yield of *V. natriegens* DSM759 Δ*dns* (wt) and *V. natriegens* DSM759 Δ*dns* Δ*lysC2* harboring the pMBI_*Vn.dapA1*:E84T vector during growth on glucose (25 mL S-limited VN medium with 20 g L^{-1} glucose and 150 mM NH_4Cl, 1 mM IPTG, 37°C, 220 rpm, n≥2).

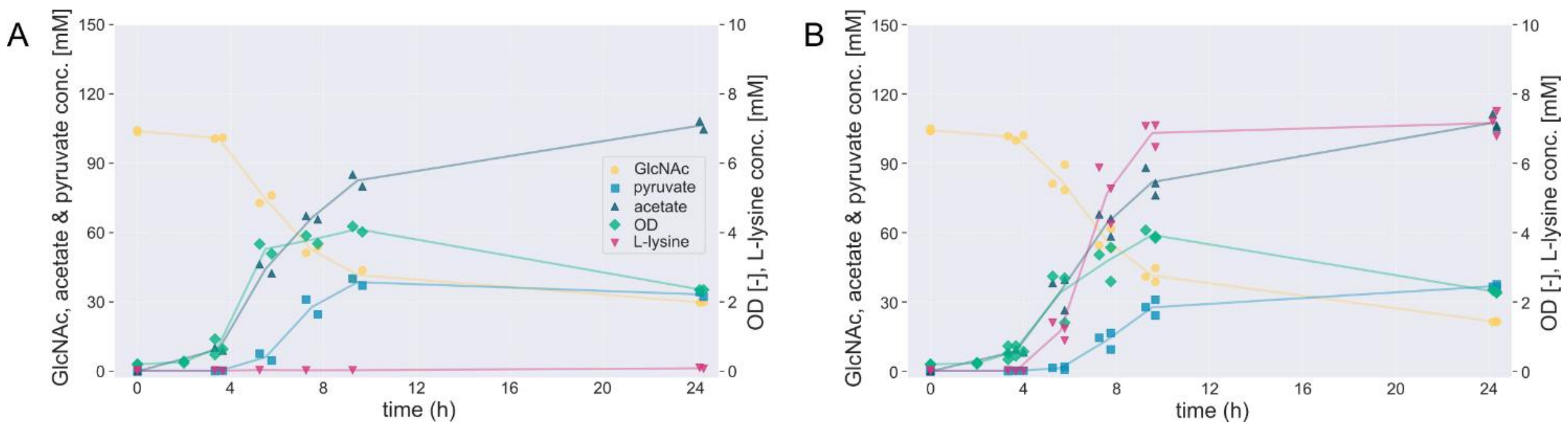

Fig. S4: Cultivation profiles of *V. natriegens* Δ*dns* harboring (A) the pMBI empty plasmid or (B) the pMBI_*Vn.lysC2_Vn.dapA1*:E84T vector on GlcNAc as carbon source. Time-course data showing biomass accumulation ($OD_{600\ nm}$), GlcNAc consumption, and formation of pyruvate, acetate, and L-lysine. Scatter points show individual raw measurements. Lines represent tolerance-based mean values, where measurements within 0.5 h were grouped and averaged to visualize the central trend without altering the original data (25 mL S-limited VN medium with 100 mM GlcNAc and 150 mM NH_4Cl, 1 mM IPTG, 37°C, 220 rpm, n≥2).

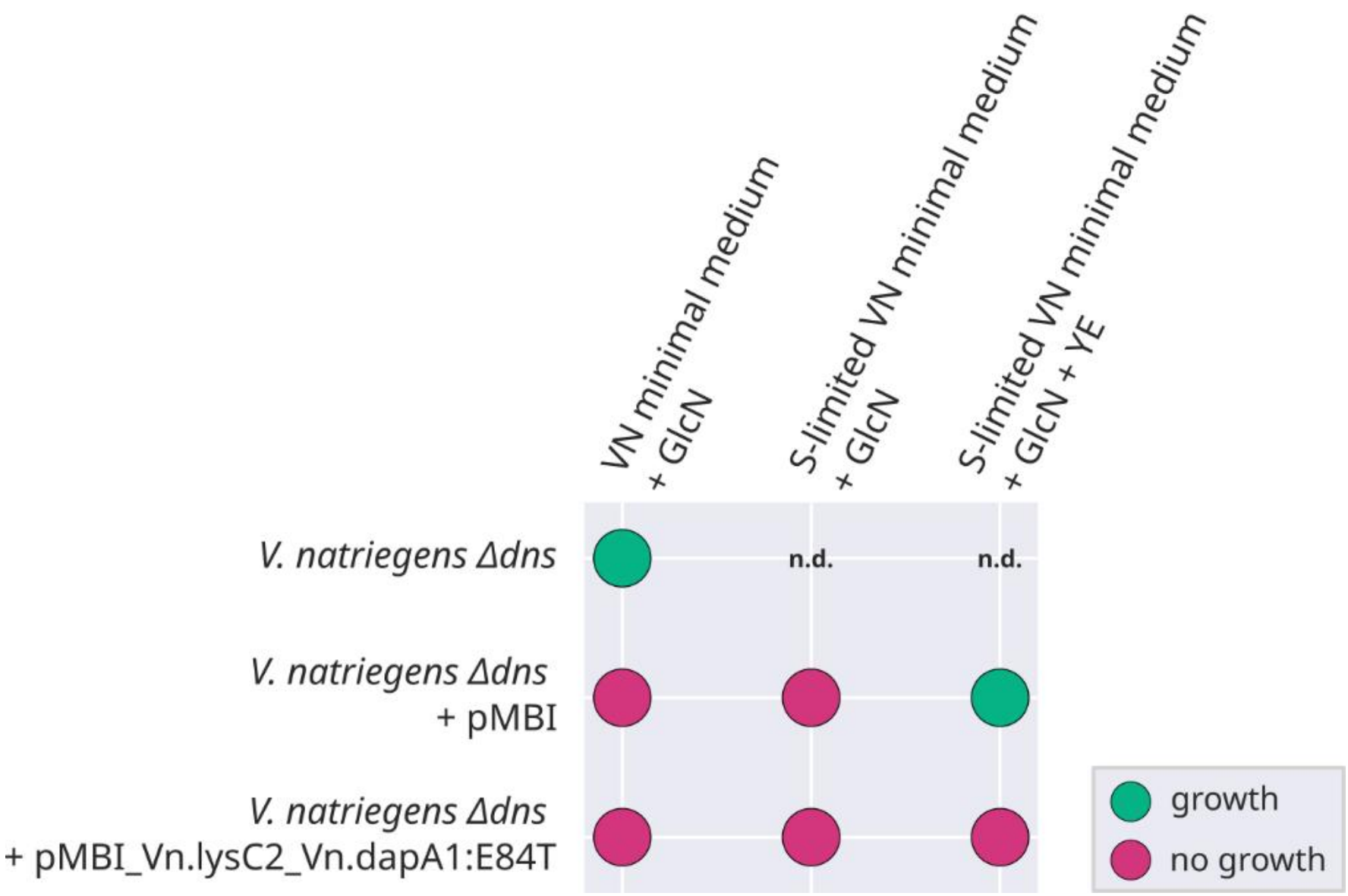

Fig. S5. Growth of *V. natriegens* Δ*dns* strains on GlcN under different cultivation conditions. Qualitative assessment of growth after 24 h of cultivation of *V. natriegens* Δ*dns*, the empty-vector control strain (*V. natriegens* Δ*dns* + pMBI), and the best-performing L-lysine producer strain (*V. natriegens* Δ*dns* + pMBI_*Vn.lysC2_Vn.dapA1*:E84T) on GlcN in VN minimal medium, sulphur-limited VN medium, and sulphur-limited VN medium supplemented with 2 g L^{-1} yeast extract (YE); n.d. – not determined.
